# Genomic and Phenotypic Characterization of Clostridium botulinum Isolates from an Infant Botulism Case Suggests Adaptation Signatures to the Gut

**DOI:** 10.1128/mbio.02384-21

**Published:** 2022-05-02

**Authors:** François P. Douillard, Yağmur Derman, Cédric Woudstra, Katja Selby, Tommi Mäklin, Martin B. Dorner, Harri Saxén, Brigitte G. Dorner, Hannu Korkeala, Miia Lindström

**Affiliations:** a Department of Food Hygiene and Environmental Health, Faculty of Veterinary Medicine, University of Helsinki, Helsinki, Finland; b Helsinki Institute for Information Technology HIIT, Department of Mathematics and Statistics, University of Helsinki, Helsinki, Finland; c Biological Toxins, Centre for Biological Threats and Special Pathogens, Robert Koch Institute, Berlin, Germany; d New Children’s Hospital, Pediatric Research Center, University of Helsinki and Helsinki University Hospital, Helsinki, Finland; University of Queensland

**Keywords:** *Clostridium botulinum*, botulism, ecology, genomics

## Abstract

In early life, the immature human gut microbiota is prone to colonization by pathogens that are usually outcompeted by mature microbiota in the adult gut. Colonization and neurotoxin production by a vegetative Clostridium botulinum culture in the gut of an infant can lead to flaccid paralysis, resulting in a clinical outcome known as infant botulism, a potentially life-threatening condition. Beside host factors, little is known of the ecology, colonization, and adaptation of C. botulinum to the gut environment. In our previous report, an infant with intestinal botulism was shown to be colonized by neurotoxigenic C. botulinum culture for 7 months. In an effort to gain ecological and evolutionary insights into this unusually long gut colonization by C. botulinum, we analyzed and compared the genomes of C. botulinum isolates recovered from the infant feces during the course of intoxication and isolates from the infant household dust. A number of observed mutations and genomic alterations pinpointed at phenotypic traits that may have promoted colonization and adaptation to the gut environment and to the host. These traits include motility, quorum-sensing, sporulation, and carbohydrate metabolism. We provide novel perspectives and suggest a tentative model of the pathogenesis of C. botulinum in infant botulism.

## INTRODUCTION

In early life, the richness and diversity of the gut microbiota are in a developing stage compared to that in the adult gut microbiota (1), rendering the gut of young infants vulnerable to colonization by pathogens. A major threat is the spores of neurotoxinogenic clostridia, which, once ingested, can germinate and outgrow to colonize the infant gut. Vegetative cultures of Clostridium botulinum produce the highly potent botulinum neurotoxin (BoNT), resulting in toxicoinfection, with a clinical outcome known as infant botulism. A similar condition is also occasionally reported in adults with underlying factors, such as gastrointestinal disorders, surgery, or antibiotic treatments, illustrating the important role of the gut microbiota in diseases and health (2–4). Host factors and environmental spore contamination are pivotal in infant botulism, and the etiology and pathogenesis of infant botulism have been well documented (5–7). From a microbial and ecological perspective, the source of contamination remains undetermined in most reported cases. It has been shown that geographical location, consumption of food products contaminated with spores, such as honey or infant milk formula, or potential exposure to contaminated soil, reptile pets, or dust particles may constitute a risk (6–12).

While the clinical course of infant botulism and the mode of action of BoNT have been established (13–15), little is known about the phenotypic and genotypic properties of C. botulinum in regard to its ability to colonize and persist in the infant gut, to compete with other gut bacteria for nutrients, and to potentially interact with the host. The long-term persistence of C. botulinum in the gut of some infants (16) is likely to trigger the emergence of polymorphic C. botulinum populations with altered or enhanced phenotypes favorable for survival and colonization in the gut. Such strategies could allow C. botulinum to escape the host immune response and to compete with other gut bacteria for resources. Genomic plasticity and dynamics likely contribute to gut adaptation (17, 18). We hypothesize that C. botulinum could adapt to the gut environment over time and therefore favor certain genotypes emerging *in situ* via single nucleotide polymorphisms, insertions, deletions, and chromosomal rearrangements.

Here, we built upon our previous clinical report from Finland, where a 3-month-old infant was diagnosed with infant botulism and, despite clinical recovery in 6 weeks, was colonized with toxinogenic C. botulinum culture for as long as 7 months (16). As part of the case follow-up, a large number of fecal samples from the infant as well as environmental samples from the infant household were collected for isolation of C. botulinum. Sampling and isolation were performed over a 7-month period, yielding gut isolates from the early onset of the disease (original colonizers) until complete clearance of C. botulinum (last colonizers). In the original report, a large set of C. botulinum isolates from fecal and dust samples were analyzed by amplified fragment length polymorphism (AFLP) and were shown to be closely related but not identical, suggesting a possible link between an environmental source and the disease (16) and adaptive evolution of C. botulinum in the infant gut. The relatively high number of isolates available for this particular infant botulism case provided a unique basis to further elaborate on C. botulinum pathophysiology in regard to spore germination, colonization, and persistence in the gut from an ecological and evolutionary perspective.

We analyzed and compared the genomes of C. botulinum isolates recovered over time from two distinct niches, infant feces (gut context) and dust samples (environment). This allowed us to examine gene polymorphisms, chromosomal rearrangements, and mobile element exchanges that may have occurred among the isolates within or between the niches. Marked differences between the isolates were found, and mutations as well as other genomic alterations pointed to phenotypic traits that relate to virulence, colonization, and possible adaptation to the gut environment, suggesting that the gut ecosystem may exert a selection pressure on C. botulinum over time. Our work brings novel insights into the pathogenesis of C. botulinum in the context of intestinal botulism and paves the way for development of prophylactic and therapeutic measures.

## RESULTS AND DISCUSSION

### Genome and mobilome of C. botulinum stool isolate ST7B.

**FIG 1:**
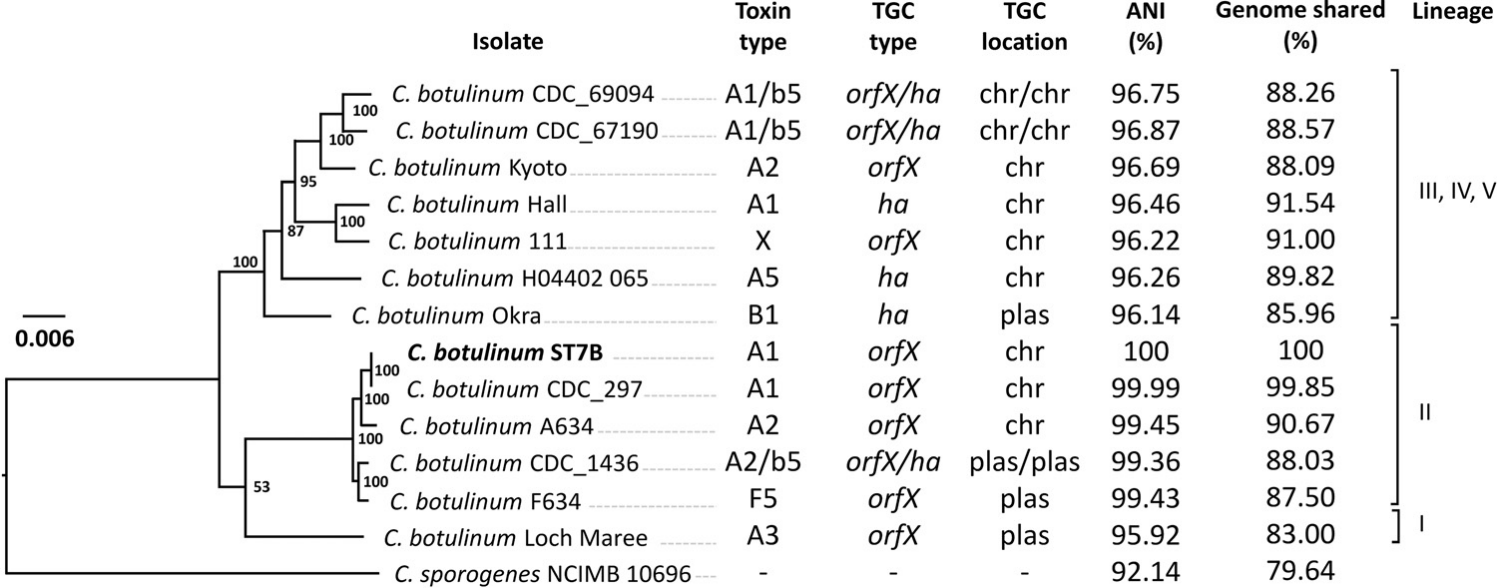
Genetic relatedness of Clostridium botulinum ST7B, isolated from infant stool, with a subset of internationally known C. botulinum group I strains using the codon tree method as described and generated in the online platform PATRIC (60). The neurotoxin type, neurotoxin gene cluster type, and location of the cluster are indicated. The strains are divided into five lineages (I to V), numbered according to a previous study (70). ANI, average nucleic acid identity; chr, chromosome; *ha*, hemagglutinin operon; *orfX*, *orfX* operon; plas, plasmid; TGC, toxin gene cluster.

C. botulinum ST7B (stool isolate, early colonizer) harbored a 3.925-Mb chromosome and a 13.75-kb plasmid with an overall G+C content of 28.16%. The genome of ST7B consisted of a total of 3,512 protein-coding sequences, 80 tRNA genes, and 27 rRNA genes. Two intact prophages and one incomplete prophage were detected. Peculiarly, C. botulinum ST7B was closely related to C. botulinum CDC_297 (GenBank version number CP006907.1), which is a historical isolate related to a foodborne botulism case (19) and has an average nucleic acid identity (ANI) of 99.99% with ST7B (Fig. 1). In line with the close phylogenetic relatedness of ST7B to CDC_297, the botulinum neurotoxin gene cluster of ST7B was similarly inserted within the *arsC* operon of the chromosome (20) and consisted of *orfX3*, *orfX2*, *orfX1*, *botR*, *p47*, *ntnh*, and *bont* (subtype A1), which is a rare cluster type for A1 strains usually harboring a hemagglutinin operon instead of an *orfX*/*p47* operon. The entire toxin gene cluster of ST7B is 100% identical to the one present in CDC_297 at the nucleotide level.

**FIG 2:**
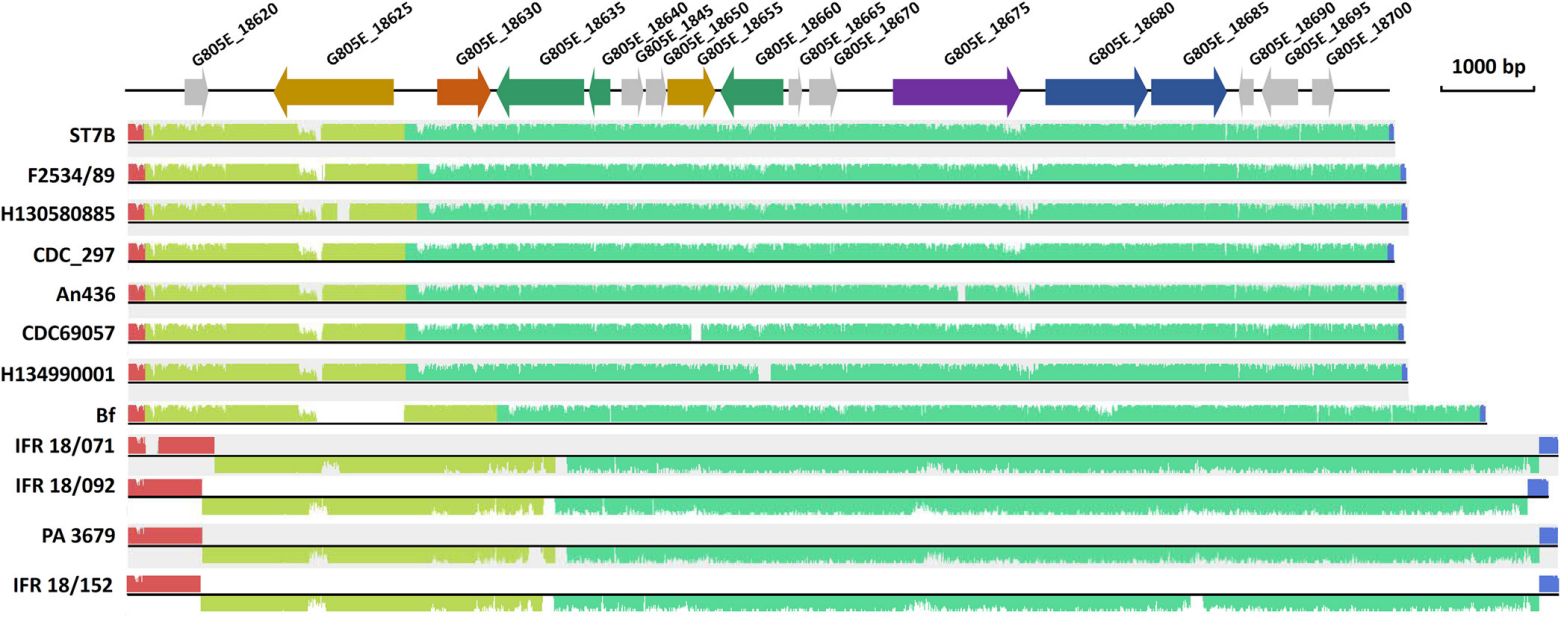
Sequence alignment of C. botulinum plasmid pST7B and related plasmids using the Mauve analysis tool. Further information relative to the degree of conservation of pST7B with homologous plasmids/contigs is shown in Table S1. Colored regions correspond to homologous regions between the different plasmid sequences. Blue arrow, gene coding for a restriction-modification system; purple arrow, gene coding for a putative replication protein; green arrow, gene coding for DNA recombination protein; yellow arrow, gene coding for amidase/*N*-acetylmuramoyl-l-alanine-amidase; orange arrow, gene coding for a putative RNA polymerase sigma factor; gray arrow, genes for coding hypothetical proteins.

The plasmid (pST7B) present in C. botulinum ST7B was 13,746 bp long and had a G+C content of 27.66%, which is slightly lower than the overall G+C content of the chromosome (28.16%). Nucleic acid BLAST analysis led to the identification of similar plasmids (or contigs) present in other group I C. botulinum and Clostridium sporogenes genomes (Fig. 2 and Table S1). These include the dual toxin type B2F5 C. botulinum strains Bf, An436, CDC69057, H130580885, and H134990001, all related to infant botulism, and also other strains, namely, CDC_297 and F2534/89. The plasmid pST7B was also related to plasmids present in *C. sporogenes* and nontoxic group I C. botulinum with an identity of ~90% and query coverage of 81% (Table S1). While most genes of pST7B were predicted to encode hypothetical proteins with unknown functions, the few annotated genes provided further insights into the potential role and function of the plasmid. First, pST7B included two genes encoding a type II restriction-modification (R/M) system. These systems are mainly known to act as a defense mechanism against foreign mobile DNA (21) and are not commonly encoded from plasmids (22). The presence of such a system may promote maintenance of pST7B in the bacteria (23) along with protecting the bacterial cells from foreign mobile DNA elements. pST7B also harbored genes encoding an *N*-acetylmuramoyl-l-alanine amidase and a putative amidase domain, whose biological function in the gut remain to be further examined. AmiC, an *N*‐acetylmuramyl‐l‐alanine amidase, was associated with motility and symbiont association by *Burkholderia* in the insect gut (24). pST7B was present in all our stool isolates (Table 1) and was also conserved to some degree in a number of other infant botulism isolates (Table S1). This leads us to hypothesize that the conserved genetic features present in pST7B and plasmids alike may play a role in colonization and persistence of C. botulinum in the infant gut. Further experimental proof of this hypothesis in an intestinal infection model is warranted.

**TABLE 1:** List of sequenced Clostridium botulinum group I type A1 isolates recovered from infant stool and environmental sources related to a previously described infant botulism case (16)

Isolate^*a*^	Origin	Sampling date	Plasmid^*b*^
H4	Household dust	13 May 2010	+
H18	Household dust	13 May 2010	+
V1	Household dust	13 May 2010	+
V4	Household dust	13 May 2010	–
V41	Household dust	13 May 2010	+
V62	Household dust	13 May 2010	+
V73	Household dust	13 May 2010	+
V134	Household dust	13 May 2010	+
V174	Household dust	13 May 2010	–
V206	Household dust	13 May 2010	+
ST25	Infant stool	9 April 2010	+
ST7B^*c*^	Infant stool	20 April 2010	+
ST19	Infant stool	21 April 2010	+
ST4	Infant stool	23 April 2010	+
ST7	Infant stool	3 May 2010	+
ST21	Infant stool	9 May 2010	+
ST29	Infant stool	14 May 2010	+
ST31	Infant stool	18 May 2010	+
ST32	Infant stool	25 May 2010	+
ST33	Infant stool	21 June 2010	+
ST34	Infant stool	19 August 2010	+
ST39	Infant stool	29 September 2010	+
ST40	Infant stool	6 October 2010	+
ST41	Infant stool	13 October 2010	+
ST43	Infant stool	28 October 2010	+
ST44	Infant stool	3 November 2010	+

a Isolates are ordered by origin and isolation date. H, environmental isolate from household dust collected from a hand held vacuum cleaner collection bag; V, environmental isolate from household dust collected from a vacuum cleaner collection bag; ST, infant stool isolate.

b +, plasmid present; –, plasmid absent; based on the present work

c Closed genome using PacBio and Illumina whole-genome sequencing. All other genomes were only sequenced by Illumina whole-genome sequencing.

10.1128/mBio.02384-21.6 TABLE S1 BLAST search results for *Clostridium botulinum* plasmid pST7B against the NCBI whole-genome shotgun contig (WGS) database (the query cover corresponds to the percentage of pST7B plasmid sequence aligned to the corresponding match; hits were ordered based on query cover and percentage identity.) Download Table S1, PDF file, 0.01 MB. Copyright © 2022 Douillard et al. 2022 Douillard et al. https://creativecommons.org/licenses/by/4.0/ This content is distributed under the terms of the Creative Commons Attribution 4.0 International license.

### Read mapping analysis singled out the isolate V174.

Read mapping of all isolates against ST7B revealed that V174 was distinct from all other isolates. While the percentage of mapped reads for the other isolates ranged from 98.29% to 99.93%, that for V174 was only 87.85% (Data Set S1). This suggested that V174 is unrelated to the other isolates, which is in line with V174 showing an AFLP profile distinct from all the other isolates related to the infant botulism case (16). Using MiGA, the closest relatives of the *de novo* assembled genome of V174 were C. botulinum AM282 (undefined origin, GenBank version number CP013683.1, 98.72% ANI) and strain BrDura (undefined origin, GenBank version number CP014151.1, 98.7% ANI). Remarkably, V174 was present in the same household as the other isolates but was not found in the infant gut, raising interesting hypotheses: (i) the infant did not get exposed to V174 spores, (ii) V174 is lacking genes or traits required for effective germination, colonization, and persistence in the infant gut, and (iii) V174 did not colonize the infant gut through competitive exclusion by the already established C. botulinum community. Our present data confirmed that V174 is unrelated to the infant botulism case, as suggested earlier (16). V174 was therefore not included in further comparative analysis.

10.1128/mBio.02384-21.5 DATA SET S1 SNPs, InDels, and structural variant calling and filtering. Download Data Set S1, PDF file, 0.5 MB. Copyright © 2022 Douillard et al. 2022 Douillard et al. https://creativecommons.org/licenses/by/4.0/ This content is distributed under the terms of the Creative Commons Attribution 4.0 International license.

### Mobile elements.

As for chromosomal regions or mobile elements present in ST7B and missing in others, the dust isolate V4 was the only one devoid of the plasmid pST7B. As hypothesized above, the absence of pST7B may be detrimental for gut persistence or colonization. No other chromosomal or mobile elements present in ST7B were missing in other sequenced isolates.

To first determine the diversity among the gut or dust isolates, we also searched for additional mobile genetic elements or chromosomal regions that were not found in our reference isolate ST7B. Reads unmapped against C. botulinum ST7B were *de novo* assembled but did not yield sequence assemblies larger than 1 kb, except in one case. In isolate V73 (dust isolate), assembly of the 1.71% unmapped reads yielded one contig of 51 kb, predicted to encode a prophage. *De novo* genome assembly showed that this 51-kb prophage-like segment was inserted within *yabG*, which encodes the sporulation-specific protease YabG (25–27). Regulation of sporulation genes by prophage disruption has been reported in other spore-formers; in Clostridioides difficile and Bacillus subtilis, cryptic prophage-encoding sequences (also called “skin,” for *sigK* intervening sequence) are inserted within *sigK* and coordinate the biosynthesis of the late sporulation sigma factor SigK during sporulation (28–30). The spore counts produced by V73 and ST7B were similar (Fig. S1). Further work on the biological function of this insertion element in *yabG* will reveal if and how it contributes to the sporulation cascade and/or the spore germination properties. It will also bring insights into the ecological relevance of prophage-bacterium interactions and prophage-mediated DNA rearrangement elements.

10.1128/mBio.02384-21.1 FIG S1 Sporulation assay of *Clostridium botulinum* isolates ST7B (purple), V1 (blue), ST34 (green), and V73 (yellow). (a) Bacteria grown in TPGY medium. (b) Bacteria grown in TPY medium. Each assay was done in triplicate (biological replicates, *n* = 3), and error bars represent the corresponding standard deviations. Download FIG S1, PDF file, 0.06 MB. Copyright © 2022 Douillard et al. 2022 Douillard et al. https://creativecommons.org/licenses/by/4.0/ This content is distributed under the terms of the Creative Commons Attribution 4.0 International license.

### C. botulinum population polymorphism may be shaped by the environment and host.

**FIG 3:**
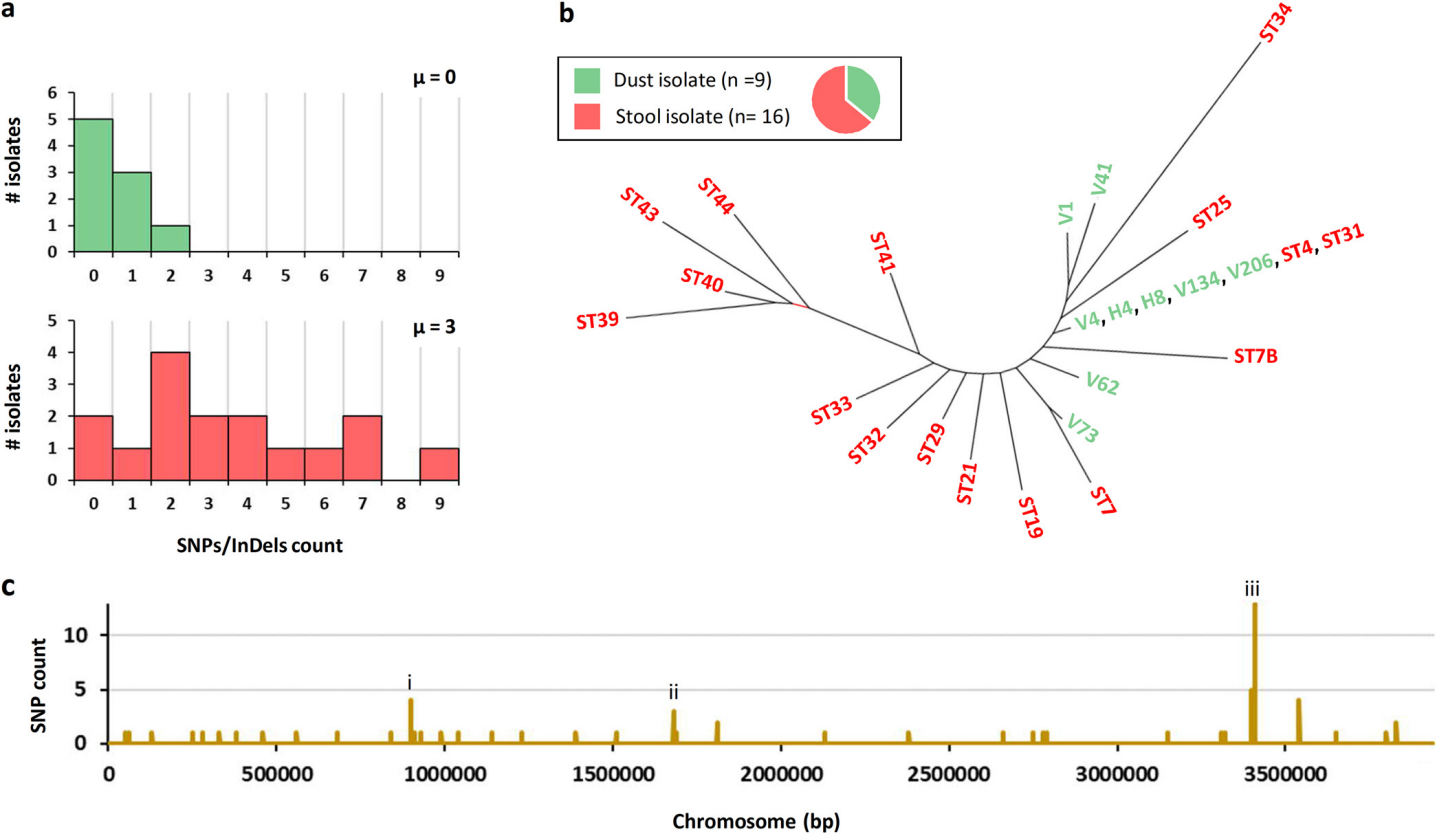
SNP analysis of all environmental (green) and stool isolates (red) of Clostridium botulinum. (a) Distribution of the isolates based on their SNP/InDel count. μ, median. (b) Neighbor-joining tree of all isolates based on Hamming distance (Saitou-Nei criterion) using PHYLOViZ software (64). (c) Chromosomal distribution of SNPs and identification of three hot spot mutation regions, labeled (i) motility (flagellar apparatus), (ii) metabolism and transport (trehalose-specific phosphotransferase system [PTS]), and (iii) quorum-sensing (*agr-2* operon).

**FIG 4:**
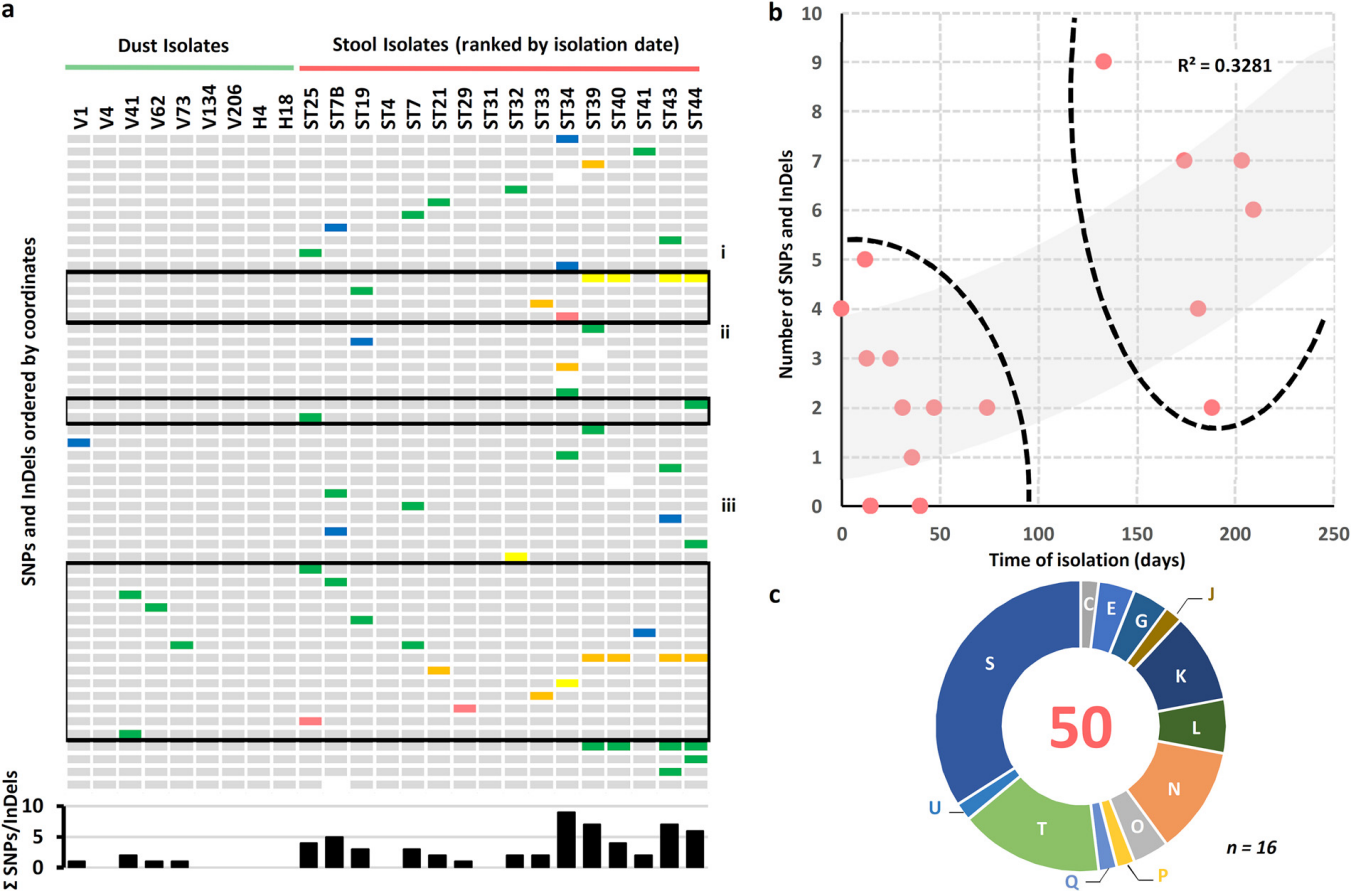
Spatial, temporal, and functional distribution of SNPs identified in isolates. (a) Heat map showing the SNPs and InDels reported for each strain. Isolates are ordered according to their isolation origin and date. White, silent mutation; blue, intergenic region; purple, sense mutation located in rRNA; green, missense mutation; orange, frameshift deletion; yellow, frameshift insertion; red, nonsense mutation. The histogram shows the total of SNPs and Indels per isolate. (b) Number of SNPs and InDels reported for each stool isolate according to the time (in days) from the isolation of the first Clostridium botulinum isolate. The two distinct clusters of stool samples are highlighted by dashed lines. The trendline (gray) and its corresponding R^2^ value are also indicated. (c) Repartition of the SNPs and InDels in stool isolates per clusters of orthologous groups of proteins (COGs) as determined using eggNOG v5.0 (71). The central number corresponds to the total count of mutated coordinates. The letter identification system describes the different COGs, as previously defined (72). *n*, number of stool isolates.

Read mapping against ST7B of 25 C. botulinum isolates related to the infant botulism case identified a number of single nucleotide polymorphisms (SNPs) and insertions/deletions (InDels) (Fig. 3 and 4, Data Set S1). SNPs present in all isolates except the reference genome ST7B were further compared with all deposited sequences. The results suggested that these SNPs emerged in the reference genome (ST7B) and not in all the other isolates. We therefore reassigned these SNPs to ST7B (Fig. 3 and 4). Interestingly, most dust isolates (H4, H8, V4, V134, V206) and two stool isolates (ST4, ST31) were identical based on SNP calling (Fig. 3). Their position within a neighbor-joining tree (Fig. 3) suggests that these isolates were the most closely related ones to the common ancestor, from which all other related isolates derived. However, we cannot conclude whether the dust isolates were the source of the infant botulism case or whether they were shed during the early phase of infection. Neither can we exclude the possibility of external sources of the infection.

The dust isolates harbored at most 2 SNPs/InDels (median μ_dust_ = 0), and stool isolates had up to 9 SNPs/InDels (median μ_stool_ = 3) (Fig. 3). Some of the isolates extracted from late stool samples (ST34, ST39, ST40, ST43, and ST44) also showed a slightly higher number of SNPs and InDels than earlier stool isolates (Fig. 4), illustrating the possible adaptive pressure exerted by the gut niche to the C. botulinum population. Most SNPs and InDels present among all isolates were found in genes relevant for pathogenesis and colonization, as further detailed below (Fig. 4 and Table S2). Specifically, three chromosomal regions were particularly prone to polymorphisms and were related to (i) quorum-sensing (*agr-2* operon), (ii) motility (flagellar apparatus), and (iii) metabolism and transport (trehalose-specific phosphotransferase system [PTS]). These systems are further discussed below. Other genes with predicted roles in gut persistence, stress resistance, or antimicrobial resistance are further detailed in Table S2. Also, a SNP present in *mutS* (DNA mismatch repair) in a late fecal isolate (ST39) may suggest the emergence of a novel lineage, potentially yielding to an even higher geno-phenotypic heterogeneity and subpopulations with enhanced adaptation or resistance to prevailing conditions (Table S2).

10.1128/mBio.02384-21.7 TABLE S2 Gene variants and potential relevance to persistence and pathogenesis (In *C. botulinum* and other bacterial species, previous studies on homologs and similarly annotated gene products may provide insights into the potential role of these genes in *C. botulinum* pathogenesis.) Download Table S2, DOCX file, 0.04 MB. Copyright © 2022 Douillard et al. 2022 Douillard et al. https://creativecommons.org/licenses/by/4.0/ This content is distributed under the terms of the Creative Commons Attribution 4.0 International license.

**FIG 5:**
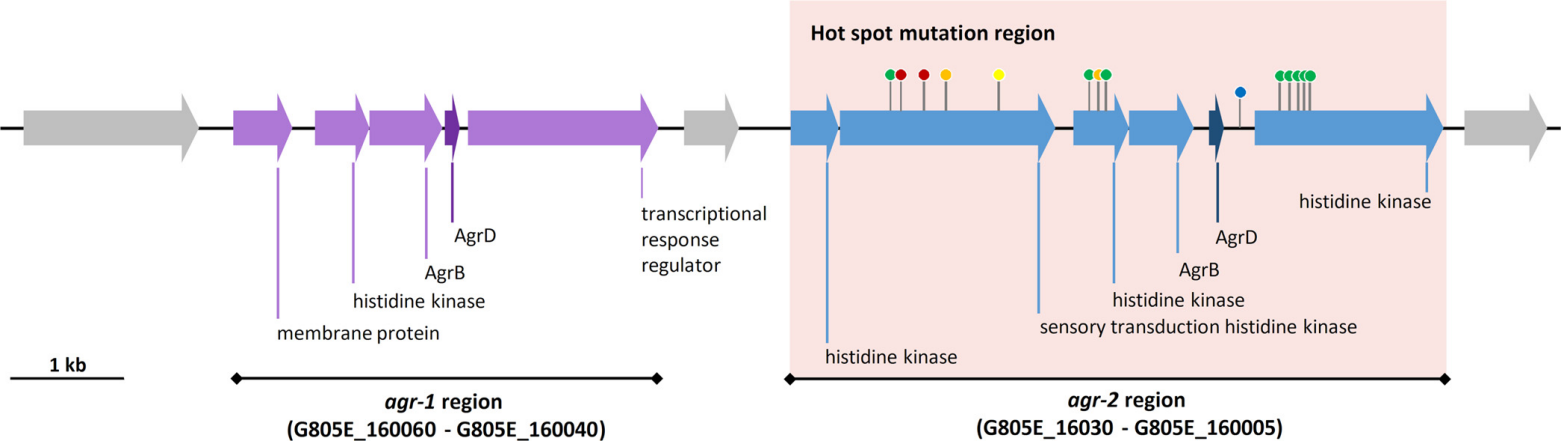
Detailed view of the *agr-1* (purple) and *agr-2* (blue) regions as predicted in the genome of Clostridium botulinum strain ST7B. Mutations detected in other isolates compared to ST7B are indicated with color-coded pins. Green, missense mutation; yellow, frameshift insertion; orange, frameshift deletion; red, nonsense mutation; blue, mutation in intergenic region.

*Agr* quorum sensing systems regulate virulence in numerous pathogens, such as C. perfringens, Enterococcus faecalis, and Staphylococcus aureus (31–33). C. botulinum ATCC 3502 has two Agr quorum sensing systems, of which the *agr-1* operon appears to have a role in sporulation control and the *agr-2* operon in positive regulation of neurotoxin production (34). Similar to ATCC 3502, ST7B and related isolates encode conserved *agr-1* and *agr-2* operons. Of all chromosomal regions, the *agr-2* operon was most prone to mutations (14 distinct locations in total), particularly in the genomes of later stool isolates (Fig. 3). The presence of nonsense mutations or frameshift mutations in the *agr-2* operon may suggest a defective or impaired Agr-2 quorum sensing system in a number of isolates (Fig. 5). In an effort to determine if these *agr-2* mutated isolates had defective toxin production, we quantified BoNT production in a subset of isolates harboring mutations in the *agr-2* operon (Fig. S2). Large differences in toxin production were observed among the isolates. However, differences in toxin production could not be solely explained by mutations located within the *agr-2* operon, suggesting that other mutations also impact neurotoxinogenesis directly or indirectly. Epigenetic factors are also likely to modulate toxinogenesis, as exemplified in ST40, where the low BoNT production could not be attributed to the presence of any particular SNPs (Fig. S2). In addition, toxin quantification of the isolates did not fit the clinical course of the infection. Indeed, the long-term colonization of the infant gut by C. botulinum was not associated with any clinical symptoms in the late time points, in line with the negative mouse bio-assays obtained from stool samples positive for C. botulinum (16). These findings suggest that toxin production of isolates grown under laboratory conditions may not truly reflect their toxinogenic phenotype in the gut environment, since exogenous or host factors may intervene in toxin regulation.

10.1128/mBio.02384-21.2 FIG S2 BoNT quantification using ELISA in *Clostridium botulinum* isolates after 24 h. (a to c) Bacteria grown in TYG (a), TPGY (b), and TPY (c). Error bars represent the minimum and maximum values obtained among replicates (*n* = 3). We selected a subset of isolates for BoNT quantification due to the presence of mutations in genes/operons that may be relevant in toxinogenesis, i.e., the *agr-2* operon. Download FIG S2, PDF file, 0.02 MB. Copyright © 2022 Douillard et al. 2022 Douillard et al. https://creativecommons.org/licenses/by/4.0/ This content is distributed under the terms of the Creative Commons Attribution 4.0 International license.

Remarkably, isolates from late fecal samples also harbored mutations in genes associated with the flagellar apparatus (*flaA*) or its biosynthesis (*flhA* and *flgN*) (Fig. 3). Flagella confer motility, known to play a role in host colonization of gut pathogens, such as C. difficile (35), and to be associated with pleiotropic effects, such as toxin production in C. difficile (36, 37). Flagella are an important colonization factor, but on the other hand, immunogenicity of the flagella may be detrimental for the bacteria in regard to the host defense system (38). Thus, bacteria have developed strategies to escape the immune response, such as direct or indirect downregulation of flagellar production (39–41). Alternatively, this might also happen by mutational emergence of isolates with flagellar defects or atypical flagellar filaments or assembly. The frameshift mutations in *flhA* and *flgN* identified in the late-colonizing C. botulinum isolates (Fig. 4 and Data Set S1) impacted motility to different degrees (Fig. S3), whereas the mutation (substitution) observed in *flaA* encoding flagellin in isolate ST19 did not alter motility. In ST19, the findings suggest that while the flagellar assembly remained intact and functional, putative alterations in the flagellin structure may have provided ecological benefits, such as escape from the host immune system or enhanced motility and chemotaxis for competing with gut microbes. The motility phenotype was associated with colony morphotype. Motile strains formed smooth-edged colonies, while nonmotile strains formed rough-edged colonies (Fig. S3 and Table S3). Similar findings relating to colony morphotype and motility behaviors have been reported in C. difficile (42).

10.1128/mBio.02384-21.3 FIG S3 Motility phenotype (a) and colony morphology (b) of *Clostridium botulinum* isolates ST7B, ST19, ST34, ST39, ST40, ST43, and ST44. Motility assays were done in triplicate. #, missense mutation in FlaA (Gln255Lys); *, nonsense mutation in FlhA (Gln327*); ¥, frame-shift insertion in FlgN (Glu3fs). Download FIG S3, PDF file, 0.2 MB. Copyright © 2022 Douillard et al. 2022 Douillard et al. https://creativecommons.org/licenses/by/4.0/ This content is distributed under the terms of the Creative Commons Attribution 4.0 International license.

10.1128/mBio.02384-21.8 TABLE S3 Colony morphotype and motility phenotype of all *Clostridium botulinum* isolatesTable S3, PDF file, 0.01 MB. Copyright © 2022 Douillard et al. 2022 Douillard et al. https://creativecommons.org/licenses/by/4.0/ This content is distributed under the terms of the Creative Commons Attribution 4.0 International license.

Trehalose transport and metabolism have been subject to intense research in regard to potential roles in C. difficile hyper-virulence (43–45), stress tolerance and antibiotic (fluoroquinolone) resistance in C. perfringens (46), and drug tolerance and resistance in Mycobacterium tuberculosis (47). In both dust and stool isolates, *treP* encoding the trehalose-specific PTS EIIBC component (transporter) was mutated at position 270 (Fig. 3). Thus, *treP* in ST25 has a threonine residue at position 270, as opposed to proline in ST7B and leucine in ST44 (most common variant). We hypothesized that this may favorably alter the phenotype of C. botulinum. While antibiotic susceptibility assays were inconclusive (Table S4), growth of ST25 and ST44 in semi-defined medium supplemented with d-trehalose suggests that these isolates utilize d-trehalose, as opposed to ST7B (Fig. S4). Interestingly, the SNP present in *treP* (position 270) of ST25 and ST44 was not isogenic based on SNP calling data (Data Set S1). We hypothesized that this may have resulted from multiple passaging of bacterial clones exogenously or from differences in selective pressure. Indeed, consecutive subculturing of isolates ST25 and ST44 in rich medium under laboratory conditions led to the changes in the *treP* SNP frequency in ST25 and ST44, suggesting that the pheno-genotype of wild isolates may be prone to further polymorphisms upon maintenance under laboratory conditions (Data Set S1). Further work with directed mutagenesis will investigate whether the different *treP* variants are associated with altered d-trehalose transport efficiency and if *treP* polymorphism is dictated by the gut environmental pressure, by providing a competitive edge.

10.1128/mBio.02384-21.4 FIG S4 Growth curves of *C. botulinum* isolates ST7B, ST25, and ST44 grown for 24 h in CDM-I medium (orange curves) and CDM-I medium supplemented with d-trehalose (blue curves). For each condition of each isolate, 12 replicates were made (four technical replicates originated from three biological replicates). Download FIG S4, PDF file, 0.1 MB. Copyright © 2022 Douillard et al. 2022 Douillard et al. https://creativecommons.org/licenses/by/4.0/ This content is distributed under the terms of the Creative Commons Attribution 4.0 International license.

10.1128/mBio.02384-21.9 TABLE S4 Antibiotic susceptibility assay-determination of MIC (μg/mL) in *Clostridium botulinum* isolates (For comparison, ST7B [reference early stool isolate] and ST41 [late stool isolate] were included along with ST25 [early stool isolate with treP variant] and ST44 [late stool isolate with treP variant]. The table shows the minimum and maximum values recorded among replicates.) Download Table S4, PDF file, 0.01 MB. Copyright © 2022 Douillard et al. 2022 Douillard et al. https://creativecommons.org/licenses/by/4.0/ This content is distributed under the terms of the Creative Commons Attribution 4.0 International license.

Along with neurotoxin production, sporulation is another key phenotypic trait in C. botulinum. In *Bacilli* and *Clostridia*, sporulation is regulated by the sporulation master switch Spo0A and involves a tightly controlled cascade of multiple sigma factors and their regulons in the mother cell and in the forespore (48). Spo0A also controls metabolic pathways and biofilm formation, and, importantly, regulates virulence in spore formers (49, 50). C. botulinum V1 and ST34 harbored mutations either upstream of *spo0A* or in its CDS, respectively. Interestingly, both strains demonstrated particularly early spore formation in two different media (Fig. S1), suggesting that these particular mutations may also impact the general physiology of the cells.

### Concluding remarks.

Here, we provide a comprehensive view of the genomic dynamics and plasticity of C. botulinum during the course of infant botulism, to further comprehend the adaptation strategies developed by C. botulinum to colonize and persist in the gut environment. While the isolates from this infant botulism case evidently shared a recent and common ancestor, marked genomic alterations were observed among the isolates. These included plasmid loss, prophage-mediated insertion element, and point mutations in genes with predicted functions in pathogenesis as well as host colonization and persistence in the gut. We also reported large variations in the phenotype of the isolates in terms of sporulation, toxin production, trehalose utilization, and motility, illustrating possible selection or adaptation to the gut conditions. While we were able to correlate genotypes and phenotypes to some extent, phenotypic heterogeneity among the isolates could not be fully associated with corresponding genotypes, suggesting that some SNPs may have remained undetected or that some phenotypic traits might result from specific SNP combinations or epigenetic factors. Nevertheless, our findings shed light on the pathogenesis of C. botulinum and led us to speculate on a two-stage model of infant botulism; upon ingestion, environmental spores with adequate phenotypic traits are first selected for their ability to survive and colonize the infant gut (stage I). This C. botulinum subpopulation is then able to germinate and outgrow, resulting in the onset of the disease. Subsequently, the population is driven toward adapting to the host gut conditions (stage II), until it reaches a tipping point where host factors and other gut microbes lead to clearance of C. botulinum. It is important to bear in mind that the phenotypic diversity observed *in vitro* may not truly reflect the phenotypic diversity of the same pool of isolates in the infant gut. Environmental and ecological conditions, i.e., pH, nutrient availability, competitive exclusion, host-bacterium cross-interactions, bacterium-bacterium interactions, and the host immune system, are likely to impact the phenotype and fitness of each isolate. In addition to DNA-level alterations, phenotypic variation is also prone to arise from epigenetic factors including gene regulation, translation, and protein-protein interactions.

This work provides novel perspectives on the pathogenesis and adaptive mechanisms of C. botulinum in toxicoinfectious botulism that may be instrumental in developing novel strategies for prevention and treatment. Comprehensive analysis of the infant gut microbiota will further refine the bacterial traits and host factors involved in the different stages of intestinal botulism in infants.

## MATERIALS AND METHODS

### Bacterial strains, genomic DNA isolation, and genome sequencing.

All C. botulinum isolates were derived from an infant botulism case previously reported by Derman et al. (16) (Table 1). Routinely, C. botulinum cultures were anaerobically grown in Trypticase-peptone-glucose-yeast extract (TPGY) broth at 37°C for 16 h, unless specified. Total genomic DNA was isolated using a Wizard genomic DNA purification kit (Promega, Wisconsin, USA) and quantified with a Qubit fluorometric assay (Thermo Scientific, Massachusetts, USA).

Isolated in the early onset of the disease (early colonizer), C. botulinum ST7B was selected as the reference genome for this work. The closed genome of ST7B was obtained using the PacBio sequencing platform (Pacific Biosciences, California, USA) at the DNA Sequencing and Genomics Laboratory (Institute of Biotechnology, University of Helsinki, Helsinki, Finland). The PacBio DNA library was constructed using the DNA template prep kit v2.0 provided by Pacific Biosciences. Genomic DNA was shredded (11-kp-long fragments) with Megaruptor (Diagenode, New Jersey, USA). The SMRTbell libraries were prepared by ligating blunt-end hairpin adapters to the fragmented DNA samples. Sequencing primers were annealed, and polymerase was bound to the SMRTbell templates using the PacBio DNA/polymerase binding kit P6. MagBeads were used to add SMRTbell libraries to SMRTcells. The PacBio RS II instrument was run for a total of 360 min.

In addition, DNA from all isolates was sent for short-read whole-genome sequencing at the Institute for Molecular Medicine Finland (FIMM), University of Helsinki (Helsinki, Finland). The Illumina sequencing library was prepared using a Nextera DNA Flex library prep kit (Illumina, California, USA), and DNA fragments of 500 to 850 bp were selected using BluePippin (Sage Science, Massachusetts, USA). Genome sequencing was performed using Illumina HiSeq 2500 or NovaSeq 6000 (Illumina) and yielded paired-end reads (2 × 101 bp).

### Genome assembly of C. botulinum ST7B.

We assembled the complete genome of ST7B using both Illumina and PacBio sequencing reads. We sketched concatenated paired-end Illumina reads with Mash (51) v2.0 (with k-mer size of 32 and minimum k-mer copy number of 3) to obtain an initial estimate for the genome size. The initial estimate was used as the target genome size parameter for Flye (52) v2.6 (with the plasmid recovery option and zero polishing iterations) to assemble the concatenated FASTQ-format PacBio subreads once. We then used the sum of the lengths of the contigs in the resulting assembly to update the target genome size parameter and reran Flye on the PacBio reads using the updated target genome size parameter, the plasmid recovery option, and 10 polishing iterations to produce the long-read-only Flye assembly. Illumina reads were aligned to the Flye assembly using the Burrows-Wheeler Aligner MEM algorithm (BWA-MEM v0.7.17-r1198-dirty) (53), and the alignment was sorted and indexed with SAMtools v1.9-63-g649f04f (54). The alignment was used to correct errors in the Flye assembly with Pilon v1.23 (55), producing the final, error-corrected assembly.

### Read mapping, SNP calling, and variant filtering.

In an effort to accurately identify SNPs and InDels, we defined a number of analytical parameters and filters most suitable for our work based on the review by Olson et al. (56). We did not expect a large number of variants, SNPs, and InDels among the isolates, which justified the need to carefully assess the quality of the variants in order to report reliable phylogenetic observations. First, rather than using a publicly available genome as a reference for read mapping, the genome of an early colonizer, namely, ST7B, was sequenced and closed (present work) to reduce any possible SNP calling bias. We suspected sequencing errors in our ST7B reference genome sequence. Therefore, we verified them by Sanger sequencing and, if confirmed, removed them from subsequent analysis (Data Set S1). Second, to mitigate errors related to sample processing, sequencing, and read mapping, all isolates described in the study were sequenced with a high sequencing depth (average mapping coverage higher than 150×) using paired-end reads (Data Set S1). Sufficient sequencing depth and the use of paired-end sequencing reads are known to reduce such errors (56). Illumina paired-end raw reads from all isolates were imported in CLC Genomics Workbench v11.0.1 (Qiagen GmbH, Germany). Reads were trimmed and mapped to the ST7B genome (Data Set S1). SNP, InDel, and structural variant calling and filtering were then carried out in CLC Genomics Workbench v11.0.1 (Data Set S1). Variants located at positions with ambiguous nonspecific read matches to the reference sequence were filtered out. We subsequently inspected variants using the CLC Genomic Workbench v11.0.1 viewer tool. The SNPs and InDels located within genes with biological relevance for this work were also confirmed by Sanger sequencing. Such genes were associated with motility, quorum sensing, sporulation, and carbohydrate transport (Data Set S1). Finally, sequencing reads with no matches to the ST7B genome were *de novo* assembled in CLC Genomics Workbench v11.0.1 with the following parameters: mapping mode = map reads back to contigs (slow), update contigs = yes, automatic bubble size = yes, minimum contig length = 1,000, automatic word size = yes, perform scaffolding = yes, auto-detect paired distances = yes, mismatch cost = 2, insertion cost = 3, deletion cost = 3, length fraction = 0.5, similarity fraction = 0.8, create list of unmapped reads = no, colorspace alignment = no, guidance only reads = no, min distance = 1, max distance = 1,000), in order to identify possible extra genetic elements present in these isolates.

### Genome assembly of V174.

In line with previous results, read mapping of the isolate V174 against ST7B revealed V174 to be distinct from the other isolates (Data Set S1). The genome of V174 was therefore assembled solely based on Illumina paired-end sequencing reads. *De novo* assembly was carried out in CLC Genomics Workbench v11.0.1 using the same parameters as listed above.

### Comparative genomic analyses.

The MiGA (Microbial Genomes Atlas) Web interface (57) was used to reveal the taxonomic identification, average nucleotide identity (ANI), and shared genome percentage of the isolates. PHASTER analysis (58, 59) was carried out to search for prophages. The genome sequence of isolate ST7B (stool) was further analyzed using the comprehensive genome analysis tool available at PATRIC (60), the RAST annotation tool kit (RASTtk) (61), the genome annotation tool Prokka (62), and the multiple-genome alignment tool Mauve v2.4.0 (63). Dendrograms and a minimum spanning tree were generated based on SNPs in each isolate using PHYLOViZ (64).

### Motility assay.

From an overnight culture in TPGY medium, C. botulinum isolates ST19, ST34, ST39, ST40, ST43, ST44, and ST7B were inoculated by stabbing wells containing 3 mL of anaerobic semisolid TPGY agar medium (0.3% wt/vol agar content) in 6-well plates. The plates were incubated anaerobically at 37°C and observed for motility after 24 h. Three biological replicate series of each isolate were included.

### Sporulation assay.

Spore counts of C. botulinum V1, ST34, V73, and ST7B cultures were determined (65) in TPGY and Trypticase-peptone-yeast extract (TPY) media after anaerobic incubation at 37°C for 24 h, 48 h, and 120 h postinoculation. For each time point, a culture aliquot was sampled and heat-treated at 80°C for 15 min. Spore counts were determined using the most-probable-number (MPN) technique as previously described (66). Three biological replicates of each isolate were included.

### Toxin quantification.

C. botulinum isolates with SNPs located within the *agr-2* operon (Data Set S1) were grown at 37°C anaerobically in either Trypticase-yeast extract d-glucose (TYG) medium, Trypticase-peptone-yeast extract (TPY) medium, or TPY medium supplemented with 0.4% wt/vol d-glucose. Each isolate was inoculated 1/100 into 10 mL of fresh medium. Samples were collected after 24 h and stored at −80°C. Toxins present in the cultures were then quantified using a sandwich enzyme-linked immunosorbent assay (ELISA) for detection of BoNT/A as previously described (67, 68). Three biological replicates of each isolate were included.

### Antibiotic resistance assay.

C. botulinum isolates ST7B (reference early isolate), ST41 (late stool isolate), ST25 (early stool isolate with *treP* SNP), and ST44 (late stool isolate with *treP* SNP) were anaerobically grown in 5 mL of TPGY medium overnight at 37°C, and 100 μL of culture was streaked onto prewarmed anaerobic bovine blood agar plates, to which Etest strips (bioMérieux, France) were applied to determine the MIC for the fluoroquinolones moxifloxacin (MX), levofloxacin (LE), and ciprofloxacin (CI), known to be linked with trehalose metabolism (46, 47). Three biological replicates were included. The MIC values were determined after 24-h incubation according to the manufacturer’s protocol.

### Growth curve assay.

C. botulinum isolates ST7B (reference isolate), ST25, and ST44 were first anaerobically grown in 5 mL of TPY medium overnight. Then, we used semi-defined medium (called CDM-I), which is a medium essentially based on previous work by Whitmer and Johnson (69) where the essential amino acid mixture was replaced by Casamino Acids (10 g/L) and l-tryptophan (0.1 g/L) and also supplemented with CoCl_2_ (1,189.7 mg/200 mL) and Na_2_SeO_3_ (17.3 mg/200 mL). Each isolate was then inoculated 1/100 into CDM-I or CDM-I plus 0.4% wt/vol d-trehalose. Then, 200 μL per well was dispensed into a 96-well plate. The plate was placed into the Hidex Sense plate reader (Hidex Oy, Finland) at 37°C for 24 h under anaerobic conditions. The optical density at 600 nm was measured every 15 min. The growth curve assay was performed in triplicate (three biological replicates consisting of four technical replicates) for each condition and isolate.

### Data availability.

The sequencing reads and all associated metadata were deposited in the NCBI Sequence Read Archive (SRA) under the project number PRJNA610151 (Data Set S1). The closed genome of C. botulinum ST7B was deposited under the accession numbers CP050251 (chromosome) and CP050252 (plasmid).
