# Supplementary material for: Genomic and Phenotypic Characterization of Clostridium botulinum Isolates from an Infant Botulism Case Suggests Adaptation Signatures to the Gut
Source: mBio. 2022 May 2;13(3):e02384-21. doi: 10.1128/mbio.02384-21 (PMC9239077; doi:10.1128/mbio.02384-21)
Supplement: TABLE S1 [file mbio.02384-21-s0007.pdf]

Table S1. BLAST search results for *Clostridium botulinum* plasmid pST7B against NCBI whole-genome shotgun contig (wgs) database. The query cover corresponds to the percentage of pST7B plasmid sequence aligned to the corresponding match. Hits were ordered based on query cover and percentage identity.

Accession number [a]	Bacterial species	Strain name	Contig name	Source	Toxin type	Total score	Query Cover	E value	Percentage Identity
ABDP01000035.1	*C. botulinum*	Bf	gcontig_1105471839388	infant botulism case	B5F2	27105	100 %	0	99.83 %
SGJI01000030.1	*C. botulinum*	F2534/89	contig00030	food-borne botulism	B5F2	25530	100 %	0	99.85 %
SGKF01000060.1	*C. botulinum*	H130580885	contig00060	infant botulism case	B5F2	25524	100 %	0	99.84 %
AQPU01000117.1[a]	*C. botulinum*	CDC297	CFSAN002368_113	-	A1	17631	100 %	0	99.71 %
AQPU01000157.1[a]	*C. botulinum*	CDC297	CFSAN002368_175	-	A1	-	-	-	-
LFON01000064.1	*C. botulinum*	An436	10258_64	infant botulism case	B5F2	25449	100 %	0	99.86 %
QVAH01000178.1	*C. botulinum*	CDC69057	NODE_96	infant botulism case	B5F2	25221	100 %	0	99.49 %
SGNM01000073.1	*C. botulinum*	H134990001	contig00073	infant botulism case	B5F2	25524	100 %	0	99.94 %
SWXE01000050.1	*C. botulinum*	IFR 18/152	contig00050	unknown, non-toxic	-	16058	81 %	0	91.34 %
SXFK01000053.1	*C. botulinum*	IFR 18/071	contig00053	unknown, non-toxic	-	16054	81 %	0	90.62 %
SXDR01000053.1	*C. botulinum*	IFR 18/092	contig00053	unknown, non-toxic	-	16001	81 %	0	90.62 %
SXDG01000068.1	*C. botulinum*	IFR 18/099	contig00068	unknown, non-toxic	-	16001	81 %	0	90.62 %
SWXG01000041.1	*C. botulinum*	IFR 18/150	contig00041	unknown, non-toxic	-	15900	81 %	0	90.62 %
SWXF01000075.1	*C. botulinum*	IFR 18/151	contig00075	unknown, non-toxic	-	16001	81 %	0	90.62 %
SWXD01000056.1	*C. botulinum*	IFR 18/153	contig00056	unknown, non-toxic	-	16001	81 %	0	90.62 %
LLZT01000062.1	*C. sporogenes*	PA 3679 (1961-4 VT95)	contig065	spore crop isolate	-	16001	81 %	0	90.62 %
LKKY02000035.1	*C. sporogenes*	PA 3679 (Camp)	plasmid unnamed1	Campbell's Soup Company	-	16001	81 %	0	90.62 %
LJTA01000024.1	*C. sporogenes*	PA 3679 (FDA VT91)	contig024	PA 3679	-	16198	81 %	0	90.62 %
LJSZ01000012.1	*C. sporogenes*	PA 3679 (NFL VT28)	contig012	National Food Laboratory	-	16174	81 %	0	90.62 %
LFVV01000039.1	*C. sporogenes*	PA 3679 (UW)	contig_40	EA Johnson Laboratory (UW)	-	16001	81 %	0	90.62 %
SWXE01000050.1	*C. botulinum*	IFR 18/152	contig00050	unknown, non-toxic	-	16058	81 %	0	91.34 %

[a] The plasmid present in CDC297 (CFSAN002368 assembly) was split into two contigs.
