# Supplementary material for: Genomic and Phenotypic Characterization of Clostridium botulinum Isolates from an Infant Botulism Case Suggests Adaptation Signatures to the Gut
Source: mBio. 2022 May 2;13(3):e02384-21. doi: 10.1128/mbio.02384-21 (PMC9239077; doi:10.1128/mbio.02384-21)

Data file S1. SNP, InDels and structural variant calling and filtering

Part A. Workflow, accession numbers and read mapping statistics

The data were deposited in NCBI under the project number PRJNA610151.

Workflow

i. Import reads

Version: CLC Genomics Workbench 11.0.1
Discard read names = No
Discard quality scores = No
Paired orientation = Paired reads (forward-reverse)
Minimum distance = 1
Maximum distance = 1000
Quality score = NCBI/Sanger or Illumina Pipeline 1.8 and later
Remove failed reads = Yes
MiSeq de-multiplexing = No
Join reads from different lanes = No

ii. Trim reads

Version: CLC Genomics Workbench 11.0.1
Quality trim = Yes
Quality limit = 0.05
Ambiguous trim = Yes
Ambiguous limit = 2
Automatic read-through adapter trimming = Yes
Remove 5' terminal nucleotides = Yes
Number of 5' terminal nucleotides = 20
Remove 3' terminal nucleotides = No
Discard short reads = No
Discard long reads = No
Save discarded sequences = No
Save broken pairs = No
Create report = Yes

iii. Map Reads to Reference - Parameters:

Version: CLC Genomics Workbench 11.0.1
References = plasmid, chromosome
Masking mode = No masking
Match score = 1
Mismatch cost = 2
Cost of insertions and deletions = Linear gap cost
Insertion cost = 3
Deletion cost = 3
Length fraction = 0.5
Similarity fraction = 0.8
Global alignment = No
Auto-detect paired distances = Yes
Non-specific match handling = Map randomly
Output mode = Create stand-alone read mappings
Create report = Yes
Collect un-mapped reads = Yes

Accession numbers and read mapping statistics

Sample	Origin	BioSample number	Sequence read archive (SRA)	Total reads	Mapped reads	Unmapped reads	% mapped reads	% unmapped reads	Average mapping coverage	% positions with less than 50 X coverage	% positions with 0-9 X coverage	Number of positions with 0-9 X coverage
H4	Dust	SAMN14278149	SRR11249086	12,821,964	12,802,130	19,834	99.85%	0.15%	260.98	0.05	0.002	89
H18	Dust	SAMN14278150	SRR11249085	12,277,424	12,244,591	32,833	99.73%	0.27%	249.65	0.20	0.002	87
V41	Dust	SAMN14278151	SRR11249074	14,297,548	14,264,721	32,827	99.77%	0.23%	290.82	0.02	0.002	86
V62	Dust	SAMN14278152	SRR11249067	12,396,848	12,369,354	27,494	99.78%	0.22%	251.45	0.05	0.002	90
V134	Dust	SAMN14278153	SRR11249066	12,917,388	12,890,317	27,071	99.79%	0.21%	262.36	0.15	0.002	90
V206	Dust	SAMN14278154	SRR11249065	15,095,730	15,056,711	39,019	99.74%	0.26%	306.92	<0.01	0.002	89
V1	Dust	SAMN14278155	SRR11249064	13,867,882	13,833,435	34,447	99.75%	0.25%	282.01	<0.01	0.002	89
V4	Dust	SAMN14278156	SRR11249063	14,601,868	14,561,491	40,377	99.72%	0.28%	296.87	1.15	0.351	13831
V73	Dust	SAMN14278157	SRR11249062	11,413,298	11,218,179	195,119	98.29%	1.71%	229.69	0.15	0.002	94
V174	Dust	SAMN14278158	SRR11249061	14,490,576	12,729,963	1,760,613	87.85%	12.15%	257.49	10.80	10.416	410225
ST25	Stool	SAMN14278159	SRR11249084	8,450,202	8,433,729	16,473	99.81%	0.19%	172.65	2.93	0.004	146
ST7B	Stool	SAMN14278160	SRR11249083	14,461,402	14,448,823	12,579	99.91%	0.09%	295.80	<0.01	0.002	87
ST19	Stool	SAMN14278161	SRR11249082	13,183,196	13,173,044	10,152	99.92%	0.08%	269.99	<0.01	0.002	87
ST4	Stool	SAMN14278162	SRR11249081	14,830,294	14,815,575	14,719	99.90%	0.10%	303.57	0.08	0.002	91
ST7	Stool	SAMN14278163	SRR11249080	12,262,802	12,252,509	10,293	99.92%	0.08%	250.99	<0.01	0.002	93
ST21	Stool	SAMN14278164	SRR11249079	12,133,444	12,124,584	8,860	99.93%	0.07%	248.54	0.04	0.002	86
ST29	Stool	SAMN14278165	SRR11249078	14,664,944	14,653,413	11,531	99.92%	0.08%	300.34	<0.01	0.002	92
ST31	Stool	SAMN14278166	SRR11249077	16,020,442	16,007,164	13,278	99.92%	0.08%	327.99	<0.01	0.002	92
ST32	Stool	SAMN14278167	SRR11249076	12,357,848	12,348,839	9,009	99.93%	0.07%	253.09	<0.01	0.002	86
ST33	Stool	SAMN14278168	SRR11249075	11,310,712	11,301,482	9,230	99.92%	0.08%	231.55	<0.01	0.002	87
ST34	Stool	SAMN14278169	SRR11249073	11,239,756	11,218,035	21,721	99.81%	0.19%	229.66	1.22	0.003	129
ST39	Stool	SAMN14278170	SRR11249072	11,052,090	11,043,792	8,298	99.92%	0.08%	226.28	0.02	0.002	82
ST40	Stool	SAMN14278171	SRR11249071	11,843,548	11,833,291	10,257	99.91%	0.09%	242.34	<0.01	0.002	81
ST41	Stool	SAMN14278172	SRR11249070	12,792,602	12,782,350	10,252	99.92%	0.08%	261.97	0.01	0.002	97
ST43	Stool	SAMN14278173	SRR11249069	11,236,728	11,227,866	8,862	99.92%	0.08%	230.07	0.35	0.002	90
ST44	Stool	SAMN14278174	SRR11249068	10,329,876	10,308,400	21,476	99.79%	0.21%	211.05	0.13	0.002	97

Remarks:

- *The isolate V4 is devoid of the 13-kb plasmid, hence the higher number of positions with no/low coverage, corresponding to the plasmid loss.*
- *The higher percentage (%) of unmapped reads in V73 reflects the presence of an additional phage inserted within the chromosome.*
- *The read mapping results of V174 against ST7B reference genome confirmed that V174 is a phylogenetically distinct isolate (strain) than all others associated with the infant botulism case, in line with our previous observations by Derman et al. (2014).*

Part B. Basic variant detection and InDels and structural variants

Basic variant detection

Version: CLC Genomics Workbench 11.0.1
Ploidy = 1
Ignore positions with coverage above = 100,000
Restrict calling to target regions = Not set
Ignore broken pairs = Yes
Ignore non-specific matches = Reads
Minimum coverage = 10
Minimum count = 2
Minimum frequency (%) = 70.0
Base quality filter = Yes
Neighbourhood radius = 5
Minimum central quality = 20
Minimum neighbourhood quality = 15
Read direction filter = No
Relative read direction filter = Yes
Significance (%) = 1.0
Read position filter = No
Remove pyro-error variants = No
Create annotated table = Yes

InDels and Structural Variants

Version: CLC Genomics Workbench 11.0.1
P-Value threshold = 0.0001
Maximum number of mismatches = 3
Minimum quality score = 30
Minimum relative consensus coverage = 0.7
Filter variants = Yes
Ignore broken pairs = Yes
Restrict calling to target regions = Not set
Create report = Yes
Create breakpoints = Yes
Create InDel variants Yes
Create structural variations Yes

Additional filtering criteria for Indels and Structural Variants

Breakpoint output:	*Fraction of non-perfectly mapped higher than 0.7*
InDel variant output:	*Variant ratio higher than 0.7*
Structural variant output:	*Variant ratio higher than 0.7*

Indels and Structural Variants were then manually curated, *i.e.* ambiguous regions, non-specific regions, low complexity or presence of inversion repeats.

Sanger sequencing: Regions of interest were amplified as per manufacturer's protocol using the HotStarTaq Master Mix Kit (Qiagen) and sent for Sanger sequencing to Macrogen, Inc. (South Korea). For each SNP are indicated the primer pair sequences. Oligonucleotide primers were ordered from Metabion GmbH (Germany).

Part C. Summary table of SNP, InDels and other structural variants

	Coordinate	V1	V4	V41	V62	V73	V134	V206	H4	H18	ST25	ST7B	ST19	ST4	ST7	ST21	ST29	ST31	ST32	ST33	ST34	ST39	ST40	ST41	ST43	ST44	DNA change	AA change	Locus tag	Predicted gene product	COG	COG categories	COG description
SNP variants	48592-chr	0	0	0	0	0	0	0	0	0	0	0	0	0	0	0	0	0	0	0	1	0	0	0	0	0	T → C	na	-	Intergenic region, upstream 30S ribosomal protein S10 (RpsJ)	na	na	na
	57420-chr	0	0	0	0	0	0	0	0	0	0	0	0	0	0	0	0	0	0	0	0	0	0	1	0	0	G → A	Val1Met	RpmD	50S ribosomal protein L30	COG1841	J	Translation, ribosomal structure and biogenesis
	127629-chr	0	0	0	0	0	0	0	0	0	0	0	0	0	0	0	0	0	0	0	0	1	0	0	0	0	delA	Lys258fs	G8E05_00715	PDZ domain-containing protein	COG3031	U	Intracellular trafficking, secretion, and vesicular transport
	247748-chr	0	0	0	0	0	0	0	0	0	0	0	0	0	0	0	0	0	0	0	1	0	0	0	0	0	G → T	silent	G8E05_01200	Pyruvate carboxylase	COG1038	C	Energy production and conversion
	284635-chr	0	0	0	0	0	0	0	0	0	0	0	0	0	0	0	0	0	1	0	0	0	0	0	0	0	G → T	Val288Leu	G8E05_01335	Membrane protein	COG3949	S	Function unknown
	332941-chr	0	0	0	0	0	0	0	0	0	0	0	0	0	0	1	0	0	0	0	0	0	0	0	0	0	G → A	Gly632Arg	lon	Endopeptidase La Type I	COG1067	O	Post-translational modification, protein turnover, and chaperon
	375390-chr	0	0	0	0	0	0	0	0	0	0	0	0	0	1	0	0	0	0	0	0	0	0	0	0	0	A → G	Glu462Gly	G8E05_01870	VWA domain-containing protein	COG2425	S	Function unknown
	460318-chr	0	0	0	0	0	0	0	0	0	0	1	0	0	0	0	0	0	0	0	0	0	0	0	0	0	A → G	na	-	Intergenic region, upstream UTP-glucose-1-phosphate uridylyltransferase Ga	na	na	na
	561576-chr	0	0	0	0	0	0	0	0	0	0	0	0	0	0	0	0	0	0	0	0	0	0	0	1	0	G → C	Val269Leu	G8E05_02745	Cell adhesion protein	ENOG502ZDXD	S	Function unknown
	678660-chr	0	0	0	0	0	0	0	0	0	1	0	0	0	0	0	0	0	0	0	0	0	0	0	0	0	G → A	Val565Ile	G8E05_03330	AAA family ATPase	COG3973	L	Replication, recombination and repair
	835081-chr	0	0	0	0	0	0	0	0	0	0	0	0	0	0	0	0	0	0	0	1	0	0	0	0	0	T → C	na	-	Intergenic region, upstream methyl-accepting chemotaxis protein	na	na	na
	900532-chr	0	0	0	0	0	0	0	0	0	0	0	0	0	0	0	0	0	0	0	0	1	1	0	1	1	insA	Glu3fs	G8E05_04245	Flagellar biosynthesis protein FlgN	COG3418	NOU	Cell motility
	910750-chr	0	0	0	0	0	0	0	0	0	0	0	1	0	0	0	0	0	0	0	0	0	0	0	0	0	C → A	Gln255Lys	G8E05_04300	Flagellin protein FlaA	COG1344	N	Cell motility
	930531-chr	0	0	0	0	0	0	0	0	0	0	0	0	0	0	0	0	0	0	1	0	0	0	0	0	0	delA	Ile611fs	G8E05_04380	DEAD/DEAH box helicase	COG0367	E	Amino acid transport and metabolism
	993298-chr	0	0	0	0	0	0	0	0	0	0	0	0	0	0	0	0	0	0	0	1	0	0	0	0	0	C → T	Gln327*	FlhA	Flagellar biosynthesis protein FlhA	COG1298	N	Cell motility
	1035690-chr	0	0	0	0	0	0	0	0	0	0	0	0	0	0	0	0	0	0	0	0	1	0	0	0	0	A → C	Lys7Gln	G8E05_04900	Aminopeptidase	COG1362	E	Amino acid transport and metabolism
	1135705-chr	0	0	0	0	0	0	0	0	0	0	0	1	0	0	0	0	0	0	0	0	0	0	0	0	0	C → A	na	-	Intergenic region, upstream asparaginase	na	na	na
	1232296-chr	0	0	0	0	0	0	0	0	0	0	0	0	0	0	0	0	0	0	0	0	1	0	0	0	0	G → A	silent	G8E05_05835	PolC-type DNA polymerase III	COG2176	L	Replication, recombination and repair
	1385000-chr	0	0	0	0	0	0	0	0	0	0	0	0	0	0	0	0	0	0	0	1	0	0	0	0	0	delT	Ile81fs	G8E05_06775	MarR family transcriptional regulator	COG1378	K	Transcription
	1509665-chr	0	0	0	0	0	0	0	0	0	0	0	0	0	0	0	0	0	0	0	1	0	0	0	0	0	A → G	silent	G8E05_07300	ABC transporter permease	COG3127	Q	Secondary metabolites biosynthesis, transport, and catabolism
	1676949-chr	0	0	0	0	0	0	0	0	0	0	0	0	0	0	0	0	0	0	0	1	0	0	0	0	0	T → C	Val82Ala	G8E05_08080	MurR/RpiR family transcriptional regulator	COG1737	K	Transcription
	1684890-chr	0	0	0	0	0	0	0	0	0	0	0	0	0	0	0	0	0	0	0	0	0	0	0	0	1	G → A	Pro270Leu	TreP	PTS system trehalose-specific EIIBC component	COG1264	G	Carbohydrate transport and metabolism
	1684891-chr	0	0	0	0	0	0	0	0	0	1	0	0	0	0	0	0	0	0	0	0	0	0	0	0	0	G → T	Pro270Thr	TreP	PTS system trehalose-specific EIIBC component	COG1264	G	Carbohydrate transport and metabolism
	1687160-chr	0	0	0	0	0	0	0	0	0	0	0	0	0	0	0	0	0	0	0	0	1	0	0	0	0	C → T	Ala195Thr	G8E05_08120	DNA mismatch repair protein MutS	COG0249	L	Replication, recombination and repair
	1813463-chr	1	0	0	0	0	0	0	0	0	0	0	0	0	0	0	0	0	0	0	0	0	0	0	0	0	G → A	na	-	Intergenic region upstream Spo0A	na	na	na
	1814301-chr	0	0	0	0	0	0	0	0	0	0	0	0	0	0	0	0	0	0	0	1	0	0	0	0	0	G → C	Trp230Cys	Spo0A	Spo0A master regulator	COG4753	T	Signal transduction mechanisms
	2128304-chr	0	0	0	0	0	0	0	0	0	0	0	0	0	0	0	0	0	0	0	0	0	0	0	1	0	T → G	Lys11Gln	G8E05_10110	ABC transporter ATP-binding protein	COG3842	P	Inorganic ion transport and metabolism
	2380101-chr	0	0	0	0	0	0	0	0	0	0	0	0	0	0	0	0	0	0	0	0	0	1	0	0	0	C → T	silent	G8E05_11315	Hypothetical protein	ENOG50302UZ	S	Function unknown
	2659033-chr	0	0	0	0	0	0	0	0	0	0	1	0	0	0	0	0	0	0	0	0	0	0	0	0	0	G → C	Val235Leu	G8E05_12575	Hypothetical protein	ENOG5030GWM	S	Function unknown
	2751196-chr	0	0	0	0	0	0	0	0	0	0	0	0	0	1	0	0	0	0	0	0	0	0	0	0	0	C → T	Gly124Arg	G8E05_12985	Putative ABC transporter permease	ENOG5033QYZ	S	Function unknown
	2783238-chr	0	0	0	0	0	0	0	0	0	0	0	0	0	0	0	0	0	0	0	0	0	0	0	1	0	A → C	na	-	Intergenic region, upstream cobyrinate a,c-diamide synthase	na	na	na
	2785098-chr	0	0	0	0	0	0	0	0	0	0	1	0	0	0	0	0	0	0	0	0	0	0	0	0	0	C → T	na	-	Intergenic region upstream transcription initiation factor TFIIIB	na	na	na
	3312248-chr	0	0	0	0	0	0	0	0	0	0	0	0	0	0	0	0	0	0	0	0	0	0	0	0	1	A → G	Val126Ala	ClpB	ATP-dependent chaperone ClpB	ENOG502NNKN	O	Post-translational modification, protein turnover, and chaperon
	3316678-chr	0	0	0	0	0	0	0	0	0	0	0	0	0	0	0	0	0	1	0	0	0	0	0	0	0	insT	Asn81fs	G8E05_15670	Helix-turn-helix transcriptional regulator	COG3800	K	Transcription
	3403617-chr	0	0	0	0	0	0	0	0	0	1	0	0	0	0	0	0	0	0	0	0	0	0	0	0	0	C → A	Lys467Asn	G8E05_16005	Histidine kinase	COG2202	T	Signal transduction mechanisms
	3403699-chr	0	0	0	0	0	0	0	0	0	0	1	0	0	0	0	0	0	0	0	0	0	0	0	0	0	C → A	Gly440Val	G8E05_16005	Histidine kinase	COG2202	T	Signal transduction mechanisms
	3403745-chr	0	0	1	0	0	0	0	0	0	0	0	0	0	0	0	0	0	0	0	0	0	0	0	0	0	C → T	Gly425Arg	G8E05_16005	Histidine kinase	COG2202	T	Signal transduction mechanisms
	3404298-chr	0	0	0	1	0	0	0	0	0	0	0	0	0	0	0	0	0	0	0	0	0	0	0	0	0	A → C	Phe240Leu	G8E05_16005	Histidine kinase	COG2202	T	Signal transduction mechanisms
	3404503-chr	0	0	0	0	0	0	0	0	0	0	0	1	0	0	0	0	0	0	0	0	0	0	0	0	0	G → T	Ser172Tyr	G8E05_16005	Histidine kinase	COG2202	T	Signal transduction mechanisms
	3405031-chr	0	0	0	0	0	0	0	0	0	0	0	0	0	0	0	0	0	0	0	0	0	0	1	0	0	C → T	na	-	Intergenic region within *agr* region II	na	na	na
	3406020-chr	0	0	0	0	1	0	0	0	0	0	0	0	0	1	0	0	0	0	0	0	0	0	0	0	0	G → T	Ser125Arg	G8E05_16020	Histidine kinase	ENOG5033NXY	S	Function unknown
	3406111-chr	0	0	0	0	0	0	0	0	0	0	0	0	0	0	0	0	0	0	0	0	1	1	0	1	1	delA	Leu95fs	G8E05_16020	Histidine kinase	ENOG5033NXY	S	Function unknown
	3406139-chr	0	0	0	0	0	0	0	0	0	0	0	0	0	0	1	0	0	0	0	0	0	0	0	0	0	A → G	Ser86Pro	G8E05_16020	Histidine kinase	ENOG5033NXY	S	Function unknown
	3406863-chr	0	0	0	0	0	0	0	0	0	0	0	0	0	0	0	0	0	0	0	1	0	0	0	0	0	insA	Gln537fs	G8E05_16025	Sensory transduction histidine kinase	COG2202	T	Signal transduction mechanisms
	3407428-chr	0	0	0	0	0	0	0	0	0	0	0	0	0	0	0	0	0	0	1	0	0	0	0	0	0	delT	Asn348fs	G8E05_16025	Sensory transduction histidine kinase	COG2202	T	Signal transduction mechanisms
	3407745-chr	0	0	0	0	0	0	0	0	0	0	0	0	0	0	0	1	0	0	0	0	0	0	0	0	0	A → C	Tyr242*	G8E05_16025	Sensory transduction histidine kinase	COG2202	T	Signal transduction mechanisms
	3407852-chr	0	0	0	0	0	0	0	0	0	1	0	0	0	0	0	0	0	0	0	0	0	0	0	0	0	G → A	Gln207*	G8E05_16025	Sensory transduction histidine kinase	COG2202	T	Signal transduction mechanisms
	3407927-chr	0	0	1	0	0	0	0	0	0	0	0	0	0	0	0	0	0	0	0	0	0	0	0	0	0	A → C	Tyr182Asp	G8E05_16025	Sensory transduction histidine kinase	COG2202	T	Signal transduction mechanisms
	3540160-chr	0	0	0	0	0	0	0	0	0	0	0	0	0	0	0	0	0	0	0	0	1	1	0	1	1	C → T	Met1Ile	G8E05_16640	DegV family protein (start codon loss)	COG1307	S	Function unknown
	3797980-chr	0	0	0	0	0	0	0	0	0	0	0	0	0	0	0	0	0	0	0	0	0	0	0	0	1	C → A	Asn448Lys	MnmE	tRNA uridine-5-carboxymethylaminomethyl(34) synthesis GTPase MnmE	COG0486	S	Function unknown
	3833093-chr	0	0	0	0	0	0	0	0	0	0	0	0	0	0	0	0	0	0	0	0	0	0	0	1	0	C → T	Thr15Ile	G8E05_18150	MarR family transcriptional regulator	ENOG502NRFU	K	Transcription
	3833247-chr	0	0	0	0	0	0	0	0	0	0	1	0	0	0	0	0	0	0	0	0	0	0	0	0	0	G → A	silent	G8E05_18150	MarR family transcriptional regulator	ENOG502NRFU	K	Transcription
Structural variants	3147761-72	0	0	0	0	0	0	0	0	0	0	0	0	0	0	0	0	0	0	0	0	0	0	0	0	1	del 12-nt	del12AA	G8E08_14935	truncated (2Fe-2S)-binding protein	ENOG5032G7V	S	Function unknown
	3646760-7	0	0	0	0	1	0	0	0	0	0	0	0	0	0	0	0	0	0	0	0	0	0	0	0	0	na	na	na	prophage inesrtion element	na	na	na
	pST7B	0	1	0	0	0	0	0	0	0	0	0	0	0	0	0	0	0	0	0	0	0	0	0	0	0	del pST7B	na	na	pST7B plasmid loss	na	na	na

Legend: na. not applicable; grey, silent mutation; blue, intergenic region; green, missense mutation; orange, frameshift deletion; yellow, frame-shift insertion; red, non-sense mutation.

Part D. Detailed re-sequencing results

The lists of variants of each isolate are detailed below. The lists combined the results from the basic variant detection tool and the InDel and structural variant tool.

Isolate ST7B (used as the reference strain in this study)

- *List of variants, insertions and deletions*

Mapping	Reference Position	Type	Reference	Allele	Count	Coverage	Frequency	Forward/reverse balance	Average quality†	Annotations	Coding region change	AA change	Remarks
plasmid	1287 (#)	Insertion	-	CTAG	1995	2295.00	86.93	0.28	36.30				FP; Sequencing errors
chromosome	2144663 (#)	Deletion	T	-	219	229.00	95.63	0.46	34.83				FP; Sequencing errors
chromosome	2144724 (#)	Deletion	T	-	175	204.00	85.78	0.35	35.05				FP; Sequencing errors
chromosome	3248010 (#)	Deletion	T	-	316	335.00	94.33	0.31	33.78	G8E05_15385	c.28delA	p.Ile10fs	FP; Sequencing errors
chromosome	3248172 (#)	Deletion	T	-	257	270.00	95.19	0.38	35.19				FP; Sequencing errors
chromosome	3368968 (#)	Deletion	GTG	-	295	330.00	89.39	0.30	34.62				FP; Sequencing errors
chromosome	3735024 (#)	Deletion	C	-	48	48.00	100.00	0.00	33.73	rRNA: G8E05_17585			Non-specific read matches

Note: FP, false positive; (#), verified by Sanger sequencing, † Phred score

- *Ambiguous variants*

Mapping	Reference Position	Reference	Allele	CLC Genomic Workbench	Remarks
chromosome	3735024	C	-	Non-specific and ambiguous read matches	Ambiguous variant, filtered out

- *Variants verified by Sanger sequencing*

Mapping	Reference Position	Reference	Allele	PCR primers	Sanger sequencing results
plasmid	1287	-	CTAG	for, TGG CTG GGG TGA AAA GTA TC<br>rev, TGG GGA TTA GTT GGA CTT GC	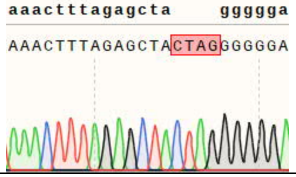

chromosome	2144663	T	-	for, CCA AGA GCT TCT CCC ATA CC<br>rev, GGA AGG AAC AAT GGC GAA T	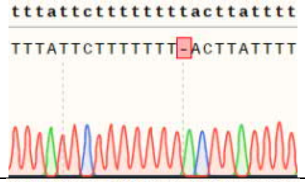
chromosome	2144724	T	-	for, ATA TAC CGC AGC CGT TTC CT<br>rev, TTT ACG CCA CAT ATC CCT ATT T	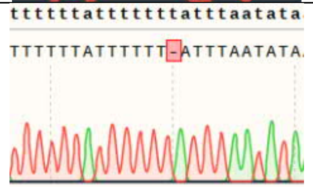
chromosome	3248010	T	-	for, CCT GTG CCA TAG CTC CAG AT<br>rev, TTG TTC CCT AAG AGT GCT AAT GC	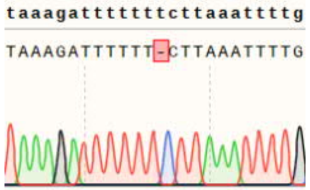
chromosome	3248172	T	-	for, CCT GTG CCA TAG CTC CAG AT<br>rev, AGA GGA AAT GGC GCT AGC TT	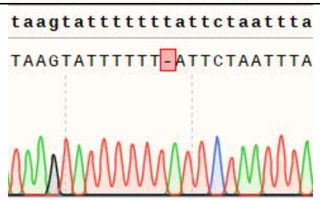
chromosome	3368968	GTG	-	for, TTG TTG AAT GCT TTT ATT ACG TAC TTT<br>rev, TTC CTG TGG AAC ACG AGA CA	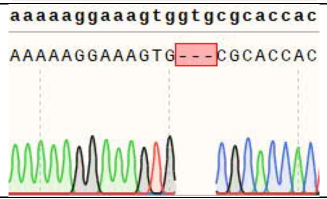

All variants reported in ST7B read mapping results are false positive (FP) and/or ambiguous variants (AV). They were either located in a region with non-specific read matches (1 occurrence) or were genome sequencing/assembly errors, as verified by Sanger sequencing above. In ST7B and other isolates, false-positive variants reported at positions 1287, 2144663, 2144724, 3248010, 3248172, 3368968 and 3735024 were therefore filtered out from the subsequent lists of variants and the downstream SNP analysis. Same applied to variants located in regions with non-specific read matches.

Isolate H4

- *List of variants*

Mapping	Reference Position	Type	Reference	Allele	Count	Coverage	Frequency	Forward/reverse read balance	Average quality†	Annotations	Coding region change	AA change
chromosome	460318	SNV	A	G	135	135	100.00	0.24	36.82			
chromosome	2659033	SNV	C	G	248	248	100.00	0.41	36.37	Gene: G8E05_12575	c.703C>G	p.Leu235Val
chromosome	2785098	SNV	C	T	171	171	100.00	0.40	35.81			
chromosome	3403699	SNV	C	A	224	224	100.00	0.30	36.20	G8E05_16005	c.1319G>T	p.Gly440Val
chromosome	3833247	SNV	A	G	293	293	100.00	0.14	36.34	G8E05_18150	c.198A>G	

† Phred score

Isolate H18

- *List of variants*

Mapping	Reference Position	Type	Reference	Allele	Count	Coverage	Frequency	Forward/reverse read balance	Average quality†	Annotations	Coding region change	AA change
chromosome	460318	SNV	A	G	119	119.00	100.00	0.17	36.60			
chromosome	2659033	SNV	C	G	217	217.00	100.00	0.47	36.12	G8E05_12575	c.703C>G	p.Leu235Val
chromosome	2785098	SNV	C	T	1750	175.00	100.00	0.44	36.31			
chromosome	3403699	SNV	C	A	209	209.00	100.00	0.26	36.66	G8E05_16005	c.1319G>T	p.Gly440Val
chromosome	3833247	SNV	A	G	253	253.00	100.00	0.16	36.10	G8E05_18150	c.198A>G	

† Phred score

Isolate V41

- *List of variants*

Mapping	Reference Position	Type	Reference	Allele	Count	Coverage	Frequency	Forward/reverse read balance	Average quality†	Annotations	Coding region change	AA change
chromosome	460318	SNV	A	G	150	150	100.00	0.21	36.60			
chromosome	2659033	SNV	C	G	299	299	100.00	0.41	36.24	G8E05_12575	c.703C>G	p.Leu235Val
chromosome	2785098	SNV	C	T	201	201	100.00	0.41	36.10			
chromosome	3403699	SNV	C	A	251	251	100.00	0.27	36.33	G8E05_16005	c.1319G>T	p.Gly440Val
chromosome	3403745	SNV	C	T	293	347	84.44	0.44	36.18	G8E05_16005	c.1273G>A	p.Gly425Arg
chromosome	3407927	SNV	A	C	81	95	85.26	0.38	36.70	G8E05_16025	c.544T>G	p.Tyr182Asp
chromosome	3833247	SNV	A	G	308	308	100.00	0.10	36.38	G8E05_18150	c.198A>G	

† Phred score

Isolate V62

- *List of variants*

Mapping	Reference Position	Type	Reference	Allele	Count	Coverage	Frequency	Forward/reverse read balance	Average quality†	Annotations	Coding region change	AA change
chromosome	460318	SNV	A	G	145	145	100.00	0.32	36.42			
chromosome	2659033	SNV	C	G	155	155	100.00	0.34	36.30	G8E05_12575	c.703C>G	p.Leu235Val
chromosome	2785098	SNV	C	T	148	148	100.00	0.42	35.54			
chromosome	3403699	SNV	C	A	132	132	100.00	0.31	35.55	G8E05_16005	c.1319G>T	p.Gly440Val
chromosome	3404298	SNV	A	C	108	108	100.00	0.44	36.00	G8E05_16005	c.720T>G	p.Phe240Leu
chromosome	3833247	SNV	A	G	329	329	100.00	0.21	36.31	G8E05_18150	c.198A>G	

† Phred score

Isolate V134

- *List of variants*

Mapping	Reference Position	Type	Reference	Allele	Count	Coverage	Frequency	Forward/reverse read balance	Average quality†	Annotations	Coding region change	AA change
chromosome	460318	SNV	A	G	119	119	100.00	0.25	36.39			
chromosome	2659033	SNV	C	G	278	278	100.00	0.43	36.18	G8E05_12575	c.703C>G	p.Leu235Val
chromosome	2785098	SNV	C	T	186	186	100.00	0.44	35.06			
chromosome	3403699	SNV	C	A	191	191	100.00	0.36	36.37	G8E05_16005	c.1319G>T	p.Gly440Val
chromosome	3833247	SNV	A	G	249	249	100.00	0.14	36.52	G8E05_18150	c.198A>G	

† Phred score

Isolate V173

- *List of variants*

Basic variant detection yielded to 91 272 variants (data not shown), indicating that both isolates (V173 and ST7B) are not closely phylogenetically related.

Subsequent *de novo* genome assembly confirmed that V173 is a distinct strain from all other isolates analysed in this work, as further elaborated in the main text of our article, which makes the listing of all variants found in V173 irrelevant. V173 was therefore not included in the SNP analysis of other isolates.

Isolate V206

- *List of variants*

Mapping	Reference Position	Type	Reference	Allele	Count	Coverage	Frequency	Forward/reverse read balance	Average quality†	Annotations	Coding region change	AA change
chromosome	460318	SNV	A	G	179	179	100.00	0.28	36.46			
chromosome	2659033	SNV	C	G	288	288	100.00	0.40	36.50	G8E05_12575	c.703C>G	p.Leu235Val
chromosome	2785098	SNV	C	T	208	208	100.00	0.41	35.79			
chromosome	3403699	SNV	C	A	246	246	100.00	0.24	36.32	G8E05_16005	:c.1319G>T	p.Gly440Val
chromosome	3833247	SNV	A	G	286	286	100.00	0.17	36.37	G8E05_18150	c.198A>G	

(#) confirmed by Sanger sequencing, † Phred score

Isolate V1

- *List of variants*

Mapping	Reference Position	Type	Reference	Allele	Count	Coverage	Frequency	Forward/reverse read balance	Average quality†	Annotations	Coding region change	AA change
chr	460318	SNV	A	G	169	169	100.00	0.26	36.43			
chr	1813463 (#)	SNV	G	A	107	113	94.69	0.35	36.44			
chr	2659033	SNV	C	G	212	212	100.00	0.46	36.15	G8E05_12575	c.703C>G	p.Leu235Val
chr	2785098	SNV	C	T	207	207	100.00	0.42	35.72			
chr	3403699	SNV	C	A	270	270	100.00	0.29	36.24	G8E05_16005	c.1319G>T	p.Gly440Val
chr	3833247	SNV	A	G	313	313	100.00	0.18	36.16	G8E05_18150	c.198A>G	

(#) verified by Sanger sequencing, † Phred score

- *Variants of verified by Sanger sequencing*

Mapping	Reference Position	Reference	Allele	PCR primers	Sanger sequencing results
chromosome	1813463	G	A	for, GCA TGA GCG GGA GTC CTA T<br>rev, TCA TCT GCG ATG ATA ACA TTG AT	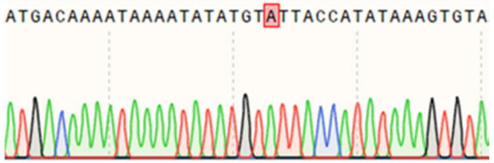

Isolate V4

- *List of variants*

Mapping	Reference Position	Type	Reference	Allele	Count	Coverage	Frequency	Forward/reverse read balance	Average quality†	Annotations	Coding region change	AA change
chromosome	460318	SNV	A	G	107	108	99.07	0.23	36.78			
chromosome	2659033	SNV	C	G	321	321	100.00	0.43	36.44	G8E05_12575	c.703C>G	p.Leu235Val
chromosome	2785098	SNV	C	T	199	199	100.00	0.41	36.16			
chromosome	3403699	SNV	C	A	175	175	100.00	0.25	36.45	G8E05_16005	c.1319G>T	p.Gly440Val
chromosome	3833247	SNV	A	G	300	300	100.00	0.15	36.52	G8E05_18150	c.198A>G	

† Phred score

- *List of Insertions, deletions and structural variants*

Mapping	Region	Type	NGS evidence	PCR Evidence
plasmid	Entire plasmid	Deletion (plasmid loss)	Coverage information<br>Read mapping results	plasmid-specific primer pairs<br>for, TGG CTG GGG TGA AAA GTA TC<br>rev, TGG GGA TTA GTT GGA CTT GC<br>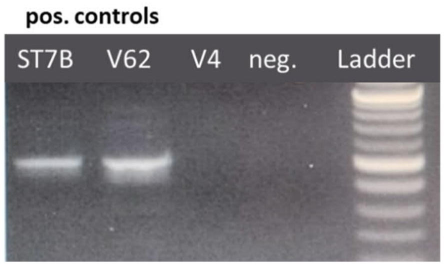

Isolate V73

- *List of variants, insertions and deletions*

Mapping	Reference Position	Type	Reference	Allele	Count	Coverage	Frequency	Forward/reverse read balance	Average quality†	Annotations	Coding region change	AA change
chromosome	460318	SNV	A	G	191	191	100.00	0.27	36.07			
chromosome	2659033	SNV	C	G	192	193	99.48	0.48	36.25	G8E05_12575	c.703C>G	p.Leu235Val
chromosome	2785098	SNV	C	T	190	190	100.00	0.42	35.80			
chromosome	3403699	SNV	C	A	117	117	100.00	0.48	36.15	G8E05_16005	c.1319G>T	p.Gly440Val
chromosome	3406020	SNV	G	T	71	94	75.53	0.40	36.18	G8E05_16020	c.375C>A	p.Ser125Arg
chromosome	3833247	SNV	A	G	218	218	100.00	0.19	35.83	G8E05_18150	c.198A>G	

† Phred score

Mapping	Region	Name	Evidence	Variant ratio	Sequence complexity	Remarks
chromosome	3646760-7	Insertion	Close breakpoints	0.92	0.252281	Presence of a large DNA element inserted into the chromosome

Note: Unmapped reads were *de novo* assembled and yielded to contigs with an estimated size of 50 kb with boundaries overlapping with the insertion region indicated above. Phaster analysis showed the presence of phage elements within the assembled fragment, suggesting that a prophage was inserted into that specific chromosomal location.

Isolate ST25

- *List of variants*

Mapping	Reference Position	Type	Reference	Allele	Count	Coverage	Frequency	Forward/reverse read balance	Average quality†	Annotations	Coding region change	AA change
chromosome	460318	SNV	A	G	42	42	100.00	0.29	36.67			
chromosome	678660	SNV	G	A	133	133	100.00	0.40	36.08	G8E05_03330	c.1693G>A	p.Val565Ile
chromosome	1684891 (#)	SNV	G	T	108	141	76.60	0.43	35.31	treP	c.808C>A	p.Pro270Thr
chromosome	2659033	SNV	C	G	143	143	100.00	0.43	36.20	G8E05_12575	c.703C>G	p.Leu235Val
chromosome	2785098	SNV	C	T	228	228	100.00	0.47	36.20			
chromosome	3403617	SNV	C	A	66	83	79.52	0.43	35.88	G8E05_16005	c.1401G>T	p.Lys467Asn
chromosome	3403699	SNV	C	A	71	71	100.00	0.49	36.01	G8E05_16005	c.1319G>T	p.Gly440Val
chromosome	3407852 (#)	SNV	G	A	40	40	100.00	0.44	34.65	G8E05_16025	c.619C>T	p.Gln207*
chromosome	3833247	SNV	A	G	99	99	100.00	0.17	36.10	G8E05_18150	c.198A>G	

† Phred score

- *Variants verified by Sanger sequencing*

Mapping	Reference Position	Reference	Allele	PCR primers	Sanger sequencing results
chromosome	1684891	G	T	for, TTC CAC CAA GTT CAG CCA TT<br>rev, AAG ATG GGC ACC ACA CAA AT	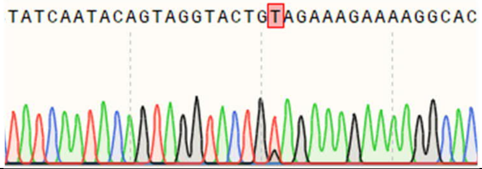
chromosome	3407852	G	A	for, CCC ATC ATA TAT AAC ATA GGT GGA TGT<br>rev, ATT GGG AAT GGA ACA TGG AA	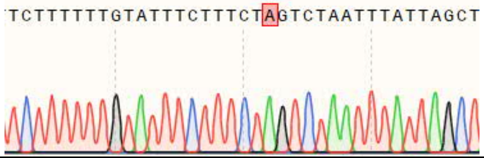

- *Stability of SNP at position 1684891 (TreP, PTS system trehalose-specific component) after 10 consecutive subculturings in TPGY (rich medium) in laboratory conditions*

ST25 was consecutively sub-cultured 10 times in TPGY broth in laboratory conditions. Using the same primer pair as indicated above, we amplified the region encompassing the TreP SNP and sent it for Sanger sequencing. We observed that the population carrying G at position 1684891 become dominant, as opposed to what we observed in the original Sanger sequencing (see above), where most of the population harboured T.

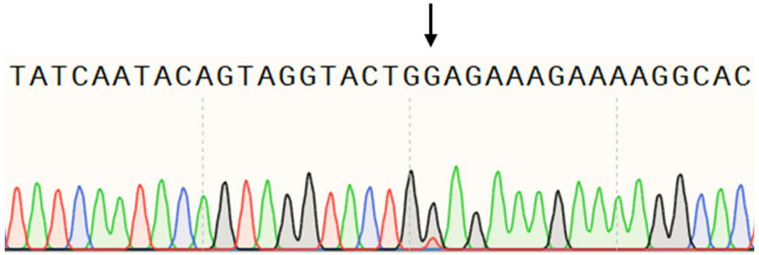

Isolate ST19

- *List of variants*

Mapping	Reference Position	Type	Reference	Allele	Count	Coverage	Frequency	Forward/reverse read balance	Average quality†	Annotations	Coding region change	AA change
chromosome	460318	SNV	A	G	143	143	100.00	0.36	36.45			
chromosome	910750 (#)	SNV	C	A	125	177	70.62	0.43	36.50	G8E05_04300	c.763C>A	p.Gln255Lys
chromosome	1135705	SNV	C	A	116	116	100.00	0.29	36.16			
chromosome	2659033	SNV	C	G	340	340	100.00	0.44	36.46	G8E05_12575	c.703C>G	p.Leu235Val
chromosome	2785098	SNV	C	T	242	242	100.00	0.46	36.30			
chromosome	3403699	SNV	C	A	235	235	100.00	0.37	36.10	G8E05_16005	c.1319G>T	p.Gly440Val
chromosome	3404503	SNV	G	T	195	205	95.12	0.28	36.41	G8E05_16005	c.515C>A	p.Ser172Tyr
chromosome	3833247	SNV	A	G	356	356	100.00	0.21	36.07	G8E05_18150	c.198A>G	

(#) verified by Sanger sequencing, † Phred score

- *Variants verified by Sanger sequencing*

Mapping	Reference Position	Reference	Allele	PCR primers	Sanger sequencing results
chromosome	910750	C	A	for, TGG AGC TGC TAG TAT TAC AGG AA<br>rev, AGA AAC TCC GCC TCC TTA CG	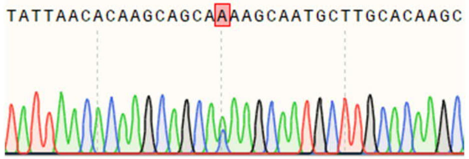

Isolate ST4

- *List of variants*

Mapping	Reference Position	Type	Reference	Allele	Count	Coverage	Frequency	Forward/reverse read balance	Average quality†	Annotations	Coding region change	Amino acid change
chromosome	460318	SNV	A	G	125	125	100	0.32	36.232			
chromosome	2659033	SNV	C	G	285	285	100	0.465035	36.46667	G8E05_12575	c.703C>G	p.Leu235Val
chromosome	2785098	SNV	C	T	302	302	100	0.44702	36.1457			
chromosome	3403699	SNV	C	A	117	117	100	0.367521	36.1453	G8E05_16005	c.1319G>T	p.Gly440Val
chromosome	3833247	SNV	A	G	288	288	100	0.138889	36.08681	G8E05_18150	c.198A>G	

† Phred score

Isolate ST7

- *List of variants*

Mapping	Reference Position	Type	Reference	Allele	Count	Coverage	Frequency	Forward/reverse read balance	Average quality†	Annotations	Coding region change	AA change
chromosome	1981	SNV	C	T	74	74	100.00	0.26	36.14	rRNA: G8E05_00010		
chromosome	375390	SNV	A	G	162	162	100.00	0.47	35.84	G8E05_01870	c.1385A>G	p.Glu462Gly
chromosome	460318	SNV	A	G	162	162	100.00	0.36	36.41			
chromosome	2659033	SNV	C	G	210	210	100.00	0.42	36.25	G8E05_12575	c.703C>G	p.Leu235Val
chromosome	2751196	SNV	C	T	209	209	100.00	0.47	36.43	G8E05_12985	c.370G>A	p.Gly124Arg
chromosome	2785098	SNV	C	T	257	257	100.00	0.43	35.98			
chromosome	3403699	SNV	C	A	166	166	100.00	0.36	36.24	G8E05_16005	c.1319G>T	p.Gly440Val
chromosome	3406020	SNV	G	T	106	145	73.10	0.48	36.83	G8E05_16020	c.375C>A	p.Ser125Arg
chromosome	3833247	SNV	A	G	303	303	100.00	0.15	36.13	G8E05_18150	c.198A>G	

† Phred score

- *Ambiguous variant – located in a region with non-specific read matches*

Mapping	Reference Position	Type	Reference	Allele	Count	Coverage	Frequency	Forward/reverse read balance	Average quality†	Annotations	Coding region change	AA change
chromosome	1981	SNV	C	T	74	74	100.00	0.26	36.14	rRNA: G8E05_00010		

Isolate ST21

- *List of variants*

Mapping	Reference Position	Type	Reference	Allele	Count	Coverage	Frequency	Forward/reverse read balance	Average quality	Annotations	Coding region change	AA change
chromosome	332941	SNV	G	A	268	268	100.00	0.38	36.62	lon	c.1894G>A	p.Gly632Arg
chromosome	460318	SNV	A	G	92	92	100.00	0.34	36.43			
chromosome	2659033	SNV	C	G	349	349	100.00	0.49	36.48	G8E05_12575	c.703C>G	p.Leu235Val
chromosome	2785098	SNV	C	T	259	259	100.00	0.40	36.40			
chromosome	3403699	SNV	C	A	169	169	100.00	0.31	35.95	G8E05_16005	c.1319G>T	p.Gly440Val
chromosome	3406139	SNV	A	G	133	133	100.00	0.25	36.43	G8E05_16020	c.256T>C	p.Ser86Pro
chromosome	3833247	SNV	A	G	210	210	100.00	0.17	36.05	G8E05_18150	c.198A>G	

† Phred score

Isolate ST29

- *List of variants*

Mapping	Reference Position	Type	Reference	Allele	Count	Coverage	Frequency	Forward/reverse read balance	Average quality†	Annotations	Coding region change	AA change
chromosome	460318	SNV	A	G	204	204	100.00	0.30	36.23			
chromosome	2659033	SNV	C	G	261	261	100.00	0.49	36.49	G8E05_12575	c.703C>G	p.Leu235Val
chromosome	2785098	SNV	C	T	256	257	99.61	0.47	36.18			
chromosome	3403699	SNV	C	A	263	263	100.00	0.41	36.29	G8E05_16005	c.1319G>T	p.Gly440Val
chromosome	3407745 (#)	SNV	A	C	133	139	95.68	0.48	36.61	G8E05_16025	c.726T>G	p.Tyr242*
chromosome	3833247	SNV	A	G	384	384	100.00	0.17	36.35	G8E05_18150	c.198A>G	

(#) verified by Sanger sequencing, † Phred score

- *Variants verified by Sanger sequencing*

Mapping	Reference Position	Reference	Allele	PCR primers	Sanger sequencing results
chromosome	3407745	A	C	for, CCC ATC ATA TAT AAC ATA GGT GGA TGT<br>rev, ATT GGG AAT GGA ACA TGG AA	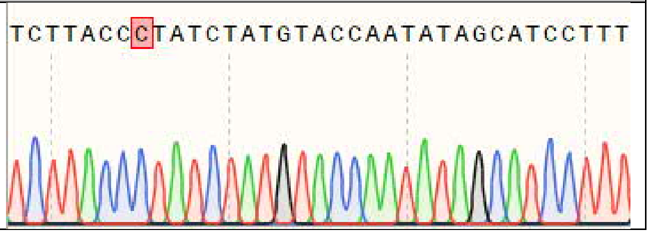

Isolate ST31

- *List of variants*

Mapping	Reference Position	Type	Reference	Allele	Count	Coverage	Frequency	Forward/reverse read balance	Average quality†	Annotations	Coding region change	AA change
chromosome	460318	SNV	A	G	198	198	100.00	0.32	36.56			
chromosome	2659033	SNV	C	G	249	249	100.00	0.43	36.38	G8E05_12575	c.703C>G	p.Leu235Val
chromosome	2785098	SNV	C	T	295	295	100.00	0.48	36.58			
chromosome	3403699	SNV	C	A	215	215	100.00	0.38	36.30	G8E05_16005	c.1319G>T	p.Gly440Val
chromosome	3833247	SNV	A	G	384	384	100.00	0.21	36.26	G8E05_18150	c.198A>G	

† Phred score

Isolate ST32

- *List of variants*

Mapping	Reference Position	Type	Reference	Allele	Count	Coverage	Frequency	Forward/reverse read balance	Average quality†	Annotations	Coding region change	AA change
chromosome	284635	SNV	G	T	246	246	100.00	0.43	36.63	G8E05_01335	c.862G>T	p.Val288Leu
chromosome	460318	SNV	A	G	171	171	100.00	0.26	36.31			
chromosome	2659033	SNV	C	G	232	232	100.00	0.49	36.35	G8E05_12575	c.703C>G	p.Leu235Val
chromosome	2785098	SNV	C	T	170	171	99.42	0.41	35.75			
chromosome	3316678	Insertion	-	T	273	283	96.47	0.38	36.45	G8E05_15670	c.248dup	p.Asn83fs
chromosome	3403699	SNV	C	A	210	210	100.00	0.34	36.33	G8E05_16005	c.1319G>T	p.Gly440Val
chromosome	3833247	SNV	A	G	340	340	100.00	0.16	35.95	G8E05_18150	c.198A>G	

† Phred score

Isolate ST33

- *List of variants*

Mapping	Reference Position	Type	Reference	Allele	Count	Coverage	Frequency	Forward/reverse read balance	Average quality†	Annotations	Coding region change	AA change
chromosome	460318	SNV	A	G	148	148	100.00	0.32	36.55			
chromosome	930531	Deletion	A	-	102	139	73.38	0.36	35.44	G8E05_04380	c.1831delA	p.Ile611fs
chromosome	2659033	SNV	C	G	184	184	100.00	0.46	36.20	G8E05_12575	c.703C>G	p.Leu235Val
chromosome	2785098	SNV	C	T	154	154	100.00	0.48	36.22			
chromosome	3403699	SNV	C	A	142	142	100.00	0.23	36.17	G8E05_16005	c.1319G>T	p.Gly440Val
chromosome	3407428 (#)	Deletion	T	-	154	164	93.90	0.42	34.30	G8E05_16025	c.1043delA	p.Asn348fs
chromosome	3833247	SNV	A	G	357	357	100.00	0.21	36.14	G8E05_18150	c.198A>G	

(#) verified by Sanger sequencing, † Phred score

- *Variant verified by Sanger sequencing*

Mapping	Reference Position	Reference	Allele	PCR primers	Sanger sequencing results
chromosome	3407428	T	-	for, GGA TGT TAC GGA TAA TGT TAT TTC CT<br>rev, TGA TGG GAT GCC AAC AAT TT	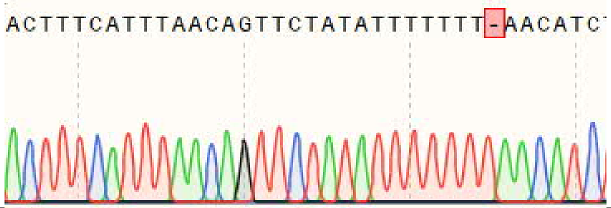

Isolate ST34

- *List of variants*

Mapping	Reference Position	Type	Reference	Allele	Count	Coverage	Frequency	Forward/reverse read balance	Average quality†	Annotations	Coding region change	AA change
chromosome	48592	SNV	T	C	325	327	99.39	0.15	35.87			
chromosome	247748	SNV	G	T	121	121	100.00	0.45	35.46	G8E05_01200	c.3375G>T	
chromosome	460318	SNV	A	G	163	163	100.00	0.29	36.41			
chromosome	835081	SNV	T	C	153	153	100.00	0.49	35.46			
chromosome	993298 (#)	SNV	C	T	291	292	99.66	0.41	35.91	flhA	c.979C>T	p.Gln327*
chromosome	1385000	Deletion	T	-	222	237	93.67	0.18	33.20	G8E05_06775	c.241delA	p.Ile81fs
chromosome	1509665	SNV	A	G	102	102	100.00	0.24	36.14	G8E05_07300	c.603A>G	
chromosome	1676949	SNV	T	C	85	85	100.00	0.45	35.07	G8E05_08080	c.245T>C	p.Val82Ala
chromosome	1814301 (#)	SNV	G	C	170	171	99.42	0.40	36.00	spo0A	c.690G>C	p.Trp230Cys
chromosome	2659033	SNV	C	G	166	166	100.00	0.42	36.22	G8E05_12575	c.703C>G	p.Leu235Val
chromosome	2785098	SNV	C	T	271	271	100.00	0.45	35.77			
chromosome	3403699	SNV	C	A	63	63	100.00	0.37	36.62	G8E05_16005	c.1319G>T	p.Gly440Val
chromosome	3406863 (#)	Insertion	-	A	59	67	88.06	0.37	36.39	G8E05_16025	c.1608dup	p.Gln537fs
chromosome	3833247	SNV	A	G	236	236	100.00	0.17	35.92	G8E05_18150	c.198A>G	

(#) verified by Sanger sequencing, † Phred score

- *Variants verified by Sanger sequencing*

Mapping	Reference Position	Reference	Allele	PCR primers	Sanger sequencing results
chromosome	993298	C	T	for, CAA TTA ACA GGA TTC CCA AAG G<br>rev, CCC ATC TCA ATA GCA CAT TGT C	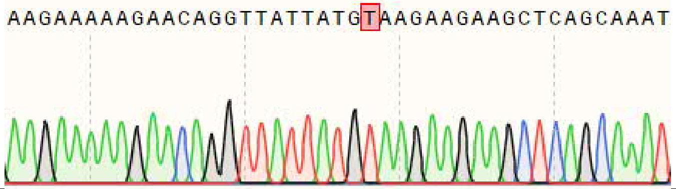

chromosome	1814301	G	C	for, GAC CAG TAG TAA TGC CGG AAA<br>rev, AAG CGA ACT GGT CAC AAA CC	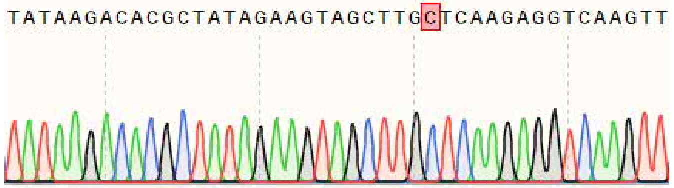
chromosome	3406863	-	A	for, CCT GCT TCT CAC AAA CTA CAG C<br>rev, TCC GTA ACA TCC TAC GCT GA	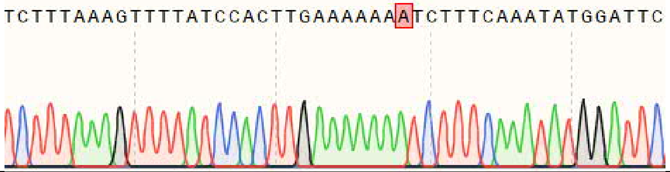

Isolate ST39

- *List of variants*

Mapping	Reference Position	Type	Reference	Allele	Count	Coverage	Frequency	Forward/reverse read balance	Average quality†	Annotations	Coding region change	AA change
chromosome	127629	Deletion	A	-	133	167	79.64	0.38	34.75	G8E05_00715	c.774delA	p.Lys258fs
chromosome	460318	SNV	A	G	98	98	100.00	0.33	36.27			
chromosome	900532 (#)	Insertion	-	A	176	178	98.88	0.50	36.47	G8E05_04245	c.6dup	p.Glu3fs
chromosome	1035690	SNV	A	C	170	170	100.00	0.31	36.59	G8E05_04900	c.19A>C	p.Lys7Gln
chromosome	1232296	SNV	G	A	150	151	99.34	0.45	36.21	G8E05_05835	c.3369G>A	
chromosome	1687160	SNV	C	T	135	141	95.74	0.31	36.21	G8E05_08120	c.583G>A	p.Ala195Thr
chromosome	2659033	SNV	C	G	315	315	100.00	0.50	36.52	G8E05_12575	c.703C>G	p.Leu235Val
chromosome	2785098	SNV	C	T	195	195	100.00	0.48	36.19			
chromosome	3403699	SNV	C	A	139	139	100.00	0.37	36.47	G8E05_16005	c.1319G>T	p.Gly440Val
chromosome	3406111 (#)	Deletion	A	-	95	112	84.82	0.26	34.52	G8E05_16020	c.284delT	p.Leu95fs
chromosome	3540160	SNV	C	T	147	147	100.00	0.39	36.66	G8E05_16640	c.3G>A	p.Met1?
chromosome	3833247	SNV	A	G	197	197	100.00	0.21	35.73	G8E05_18150	c.198A>G	

(#) verified by Sanger sequencing, † Phred score

- *Variants verified by Sanger sequencing*

Mapping	Reference Position	Reference	Allele	PCR primers	Sanger sequencing results
chromosome	900532	-	A	for, TGA AGG TAA ATG GAG TAG GTA TAA CA<br>rev, GAT TGC GTC TAG CCA TTT CC	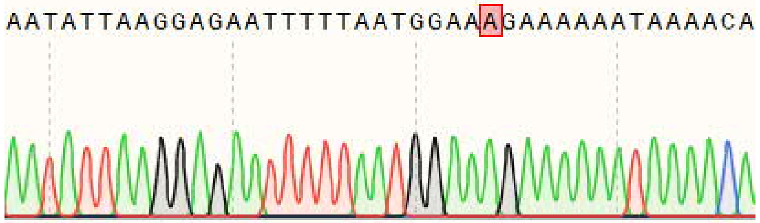

chromosome	3406111	A	-	for, TAC TGC GAA AGC ACC ATA CG<br>rev, GCA TTT AGC AAT AGT AAG ATA AGC AAA	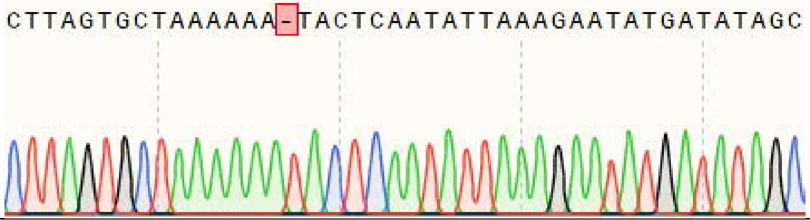

Isolate ST40

- *List of variants*

Mapping	Reference Position	Type	Reference	Allele	Count	Coverage	Frequency	Forward/reverse read balance	Average quality†	Annotations	Coding region change	AA change
chromosome	460318	SNV	A	G	100	100	100.00	0.32	36.38			
chromosome	900532 (#)	Insertion	-	A	134	136	98.53	0.47	36.55	G8E05_04245	c.6dup	p.Glu3fs
chromosome	2380101	SNV	C	T	121	121	100.00	0.44	35.81	G8E05_11315	c.21G>A	
chromosome	2659033	SNV	C	G	285	285	100.00	0.49	36.40	G8E05_12575	c.703C>G	p.Leu235Val
chromosome	2785098	SNV	C	T	131	131	100.00	0.40	35.99			
chromosome	3403699	SNV	C	A	130	130	100.00	0.35	35.65	G8E05_16005	c.1319G>T	p.Gly440Val
chromosome	3406111 (#)	Deletion	A	-	108	123	87.80	0.19	35.60	G8E05_16020	c.284delT	p.Leu95fs
chromosome	3540160	SNV	C	T	143	143	100.00	0.46	36.23	G8E05_16640	c.3G>A	p.Met1?
chromosome	3833247	SNV	A	G	471	471	100.00	0.18	36.17	G8E05_18150	c.198A>G	

(#) verified by Sanger sequencing, † Phred score

- *Variants verified by Sanger sequencing*

Mapping	Reference Position	Reference	Allele	PCR primers	Sanger sequencing results
chromosome	900532	-	A	for, TGA AGG TAA ATG GAG TAG GTA TAA CA<br>rev, GAT TGC GTC TAG CCA TTT CC	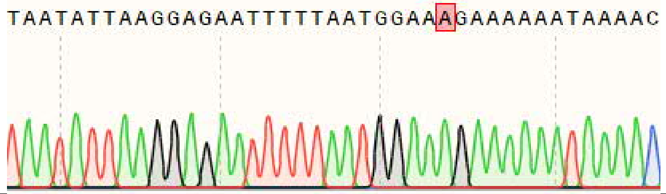
chromosome	3406111	A	-	for, TAC TGC GAA AGC ACC ATA CG<br>rev, GCA TTT AGC AAT AGT AAG ATA AGC AAA	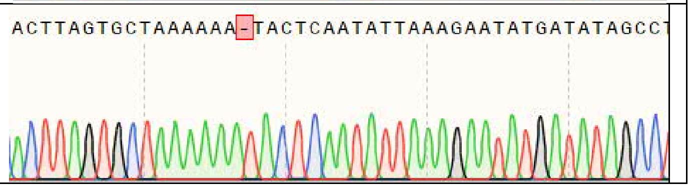

Isolate ST41

- *List of variants*

Mapping	Reference Position	Type	Reference	Allele	Count	Coverage	Frequency	Forward/reverse read balance	Average quality†	Annotations	Coding region change	AA change
chromosome	57420	SNV	G	A	260	260	100.00	0.45	36.25	rpmD	c.1G>A	p.Val1Met
chromosome	460318	SNV	A	G	116	116	100.00	0.25	36.81			
chromosome	2659033	SNV	C	G	234	235	99.57	0.44	36.15	G8E05_12575	c.703C>G	p.Leu235Val
chromosome	2785098	SNV	C	T	252	252	100.00	0.43	36.13			
chromosome	3403699	SNV	C	A	139	139	100.00	0.37	36.12	G8E05_16005	c.1319G>T	p.Gly440Val
chromosome	3405031	SNV	C	T	118	118	100.00	0.33	35.93			
chromosome	3833247	SNV	A	G	276	276	100.00	0.15	36.31	G8E05_18150	c.198A>G	

† Phred score

Isolate ST43

- *List of variants*

Mapping	Reference Position	Type	Reference	Allele	Count	Coverage	Frequency	Forward/reverse read balance	Average quality†	Annotations	Coding region change	AA change
chromosome	460318	SNV	A	G	111	111	100.00	0.37	36.50			
chromosome	561576	SNV	G	C	283	283	100.00	0.47	36.39	G8E05_02745	c.805G>C	p.Val269Leu
chromosome	900532 (#)	Insertion	-	A	256	259	98.84	0.43	36.32	G8E05_04245	c.6dup	p.Glu3fs
chromosome	2128304	SNV	T	G	104	104	100.00	0.39	36.37	G8E05_10110	c.31A>C	p.Lys11Gln
chromosome	2659033	SNV	C	G	230	230	100.00	0.50	36.43	G8E05_12575	c.703C>G	p.Leu235Val
chromosome	2783238	SNV	A	C	227	229	99.13	0.27	35.96			
chromosome	2785098	SNV	C	T	248	248	100.00	0.49	35.99			
chromosome	3403699	SNV	C	A	108	108	100.00	0.35	36.48	G8E05_16005	c.1319G>T	p.Gly440Val
chromosome	3406111 (#)	Deletion	A	-	49	56	87.50	0.24	35.00	G8E05_16020	c.284delT	p.Leu95fs
chromosome	3540160	SNV	C	T	225	225	100.00	0.40	36.59	G8E05_16640	c.3G>A	p.Met1?
chromosome	3833093	SNV	C	T	192	192	100.00	0.41	36.30	G8E05_18150	c.44C>T	p.Thr15Ile
chromosome	3833247	SNV	A	G	230	230	100.00	0.14	35.67	G8E05_18150	c.198A>G	

(#) verified by Sanger sequencing, † Phred score

- *Variants verified by Sanger sequencing*

Mapping	Reference Position	Reference	Allele	PCR primers	Sanger sequencing results
chromosome	900532	-	A	for, TGA AGG TAA ATG GAG TAG GTA TAA CA<br>rev, GAT TGC GTC TAG CCA TTT CC	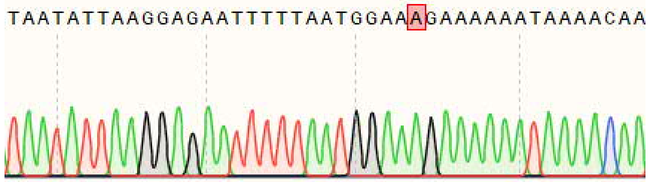
chromosome	3406111	A	-	for, TAC TGC GAA AGC ACC ATA CG<br>rev, GCA TTT AGC AAT AGT AAG ATA AGC AAA	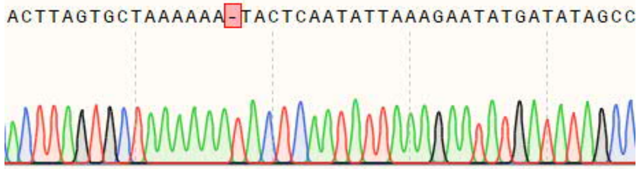

Isolate ST44

- *List of variants*

Mapping	Reference Position	Type	Reference	Allele	Count	Coverage	Frequency	Forward/reverse read balance	Average quality†	Annotations	Coding region change	AA change
chromosome	460318	SNV	A	G	181	181	100.00	0.28	36.01			
chromosome	900532 (#)	Insertion	-	A	188	191	98.43	0.42	36.10	G8E05_04245	c.6dup	p.Glu3fs
chromosome	1684890 (#)	SNV	G	A	128	132	96.97	0.38	35.58	treP	c.809C>T	p.Pro270Leu
chromosome	2659033	SNV	C	G	152	152	100.00	0.49	36.08	G8E05_12575	c.703C>G	p.Leu235Val
chromosome	2785098	SNV	C	T	233	234	99.57	0.50	35.99			
chromosome	3312248	SNV	A	G	221	221	100.00	0.35	35.92	clpB	c.377T>C	p.Val126Ala
chromosome	3403699	SNV	C	A	105	105	100.00	0.48	35.97	G8E05_16005	c.1319G>T	p.Gly440Val
chromosome	3406111 (#)	Deletion	A	-	61	66	92.42	0.15	35.05	G8E05_16020	c.284delT	p.Leu95fs
chromosome	3540160	SNV	C	T	237	237	100.00	0.42	36.27	G8E05_16640	c.3G>A	p.Met1?
chromosome	3797980	SNV	C	A	202	202	100.00	0.35	36.31	mnmE	c.1344C>A	p.Asn448Lys
chromosome	3833247	SNV	A	G	198	198	100.00	0.16	35.44	G8E05_18150	c.198A>G	

(#) verified by Sanger sequencing, † Phred score

Mapping	Region	Type	Reference	Allele	Length	Evidence	Variant ratio	Sequence complexity
chromosome	3147761-3147772	Deletion	TTTTATAGCATT	-	12	Paired breakpoint	0.994083	0.583333

Deletion was confirmed by performing de novo assembly of ST44 read sequencing and sequence alignment of the region encompassing the deletion with ST7B reference genome sequence.

- *Variants verified by Sanger sequencing*

Mapping	Reference Position	Reference	Allele	PCR primers	Sanger sequencing results
chromosome	900532	-	A	for, TGA AGG TAA ATG GAG TAG GTA TAA CA<br>rev, GAT TGC GTC TAG CCA TTT CC	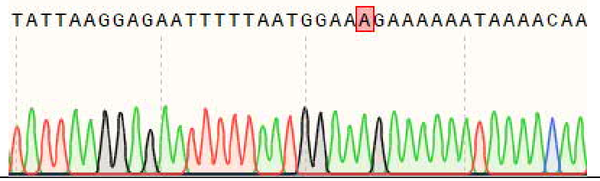

chromosome	1684890	G	A	for, TTC CAC CAA GTT CAG CCA TT<br>rev, AAG ATG GGC ACC ACA CAA AT	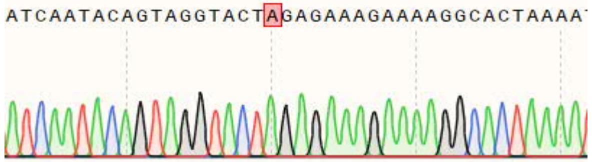
chromosome	3406111	A	-	for, TAC TGC GAA AGC ACC ATA CG<br>rev, GCA TTT AGC AAT AGT AAG ATA AGC AAA	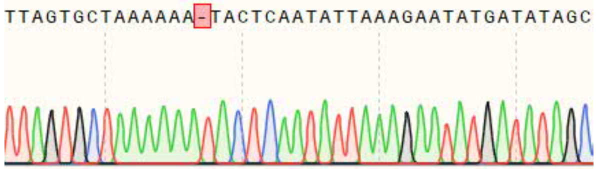

- *Stability of SNP at position 1684890 (TreP, PTS system trehalose-specific component) after 10 consecutive subculturings in TPGY (rich medium) in laboratory conditions*

ST44 was consecutively sub-cultured 10 times in TPGY broth in laboratory conditions. Using the same primer pair as indicated above, we amplified the region encompassing the TreP SNP and sent it for Sanger sequencing. We observed that the population carrying G at position 1684890 become dominant, as opposed to what we observed in the original Sanger sequencing (see above), where the whole population harboured A.

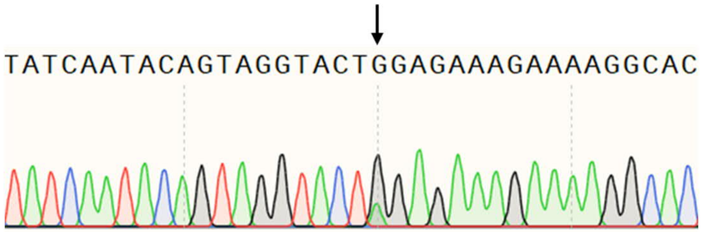

Supplement: DATA SET S1 [file mbio.02384-21-s0001.pdf]
