# Supplementary material for: Genomic and Phenotypic Characterization of Clostridium botulinum Isolates from an Infant Botulism Case Suggests Adaptation Signatures to the Gut
Source: mBio. 2022 May 2;13(3):e02384-21. doi: 10.1128/mbio.02384-21 (PMC9239077; doi:10.1128/mbio.02384-21)
Supplement: FIG S1 [file mbio.02384-21-s0002.pdf]

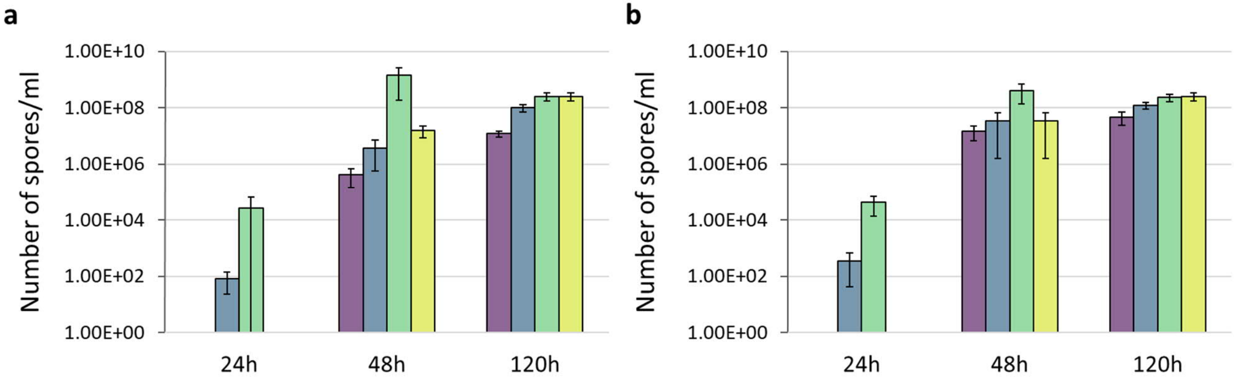

Figure S1. Sporulation assay of *Clostridium botulinum* isolates ST7B (purple), V1 (blue), ST34 (green) and V73 (yellow). (A) Bacteria grown in TPGY medium. (B) Bacteria grown in TPY medium. Each assay was done in triplicate (biological replicates, N =3) and error bars represent the corresponding standard deviations.
