# Supplementary material for: Genomic and Phenotypic Characterization of Clostridium botulinum Isolates from an Infant Botulism Case Suggests Adaptation Signatures to the Gut
Source: mBio. 2022 May 2;13(3):e02384-21. doi: 10.1128/mbio.02384-21 (PMC9239077; doi:10.1128/mbio.02384-21)
Supplement: TABLE S2 [file mbio.02384-21-s0006.docx]

**Table S2.** Gene variants and potential relevance to persistence and pathogenesis. In *C. botulinum* and other bacterial species, previous studies on homologs and similarly annotated gene products may provide insights into the potential role of these genes in *C. botulinum* pathogenesis.

| **Coordinate** | **Locus tag** | **Isolates** | **Predicted gene product** | **Potentially associated with niche persistence and/or pathogenesis** | **References** |
| --- | --- | --- | --- | --- | --- |
| 48592 | - | ST34 | upstream 30S ribosomal protein S10 (RpsJ) | tigecycline resistance | (1) |
| 57420 | RpmD | ST41 | 50S ribosomal protein L30 | - | - |
| 127636 | G8E05_00715 | ST39 | PDZ domain-containing protein | virulence | (2) |
| 247748 | G8E05_01200 | ST34 | pyruvate carboxylase | virulence | (3) |
| 284635 | G8E05_01335 | ST32 | membrane protein | cell adhesion (COG3949) | - |
| 375390 | G8E05_01870 | ST7 | VWA domain-containing protein | cell adhesion (COG2425) | - |
| 460318 | GalU | ST7B | upstream UTP-glucose-1-phosphate uridylyltransferase | virulence | (4) |
| 561576 | G8E05_02745 | ST43 | discoidin domain-containing protein | - | - |
| 678660 | G8E05_03330 | ST25 | AAA family ATPase | - | - |
| 835081 | - | ST34 | upstream methyl-accepting chemotaxis protein | chemotaxis | - |
| 900532 | G8E05_04245 | ST39, ST40, ST43, ST44 | flagellar biosynthesis protein FlgN | motility, adherence | (5) |
| 910750 | G8E05_04300 | ST19 | flagellin protein FlaA | motility, adherence | (5) |
| 930531 | G8E05_04380 | ST33 | DEAD/DEAH box helicase | - | - |
| 993298 | FlhA | ST34 | flagellar biosynthesis protein FlhA | motility, adherence | (6) |
| 1035690 | G8E05_04900 | ST39 | aminopeptidase | - | - |
| 1135705 | - | ST19 | upstream asparaginase | - | - |
| 1232296 | G8E05_05835 | ST39 | polC-type DNA polymerase III | - | - |
| 1385007 | G8E05_06775 | ST34 | MarR family transcriptional regulator | antibiotic resistance | (7) |
| 1509665 | G8E05_07300 | ST34 | ABC transporter permease | transport (PFAM02687) | - |
| 1676949 | G8E05_08080 | ST34 | MurR/RpiR family transcriptional regulator | - | - |
| 1684890 | TreP | ST44 | PTS system trehalose-specific EIIBC component | antibiotic resistance, stress resistance, others | (8-10) |
| 1684891 | TreP | ST25 | PTS system trehalose-specific EIIBC component | antibiotic resistance, stress resistance, others | (8-10) |
| 1687160 | G8E05_08120 | ST39 | DNA mismatch repair protein MutS | hypermutator lineage, antibiotic resistance | (11) |
| 1813463 | - | V1 | intergenic region upstream Spo0A | sporulation | (12) |
| 1814301 | Spo0A | ST34 | Spo0A master regulator | sporulation | (12) |
| 2128304 | G8E05_10110 | ST43 | ABC transporter ATP-binding protein | carbohydrate transport and metabolism (COG3839) | - |
| 2659033 | G8E05_12575 | ST7B | hypothetical protein | - | - |
| 2751196 | G8E05_12985 | ST7 | hypothetical protein | - | - |
| 2783238 | - | ST43 | upstream cobyrinate a,c-diamide synthase | - | - |
| 2785098 | - | ST7B | upstream transcription initiation factor TFIIIB | - | - |
| 3312248 | ClpB | ST44 | ATP-dependent chaperone ClpB | stress response, virulence, colonization | (13-15) |
| 3316678 | G8E05_15670 | ST32 | helix-turn-helix transcriptional regulator | - | - |
| 3403617 | G8E05_16005 | ST25 | histidine kinase | quorum sensing, virulence, toxin production | (16, 17) |
| 3403699 | G8E05_16005 | ST7B | histidine kinase | quorum sensing, virulence, toxin production | (16, 17) |
| 3403745 | G8E05_16005 | V41 | histidine kinase | quorum sensing, virulence, toxin production | (16, 17) |
| 3404298 | G8E05_16005 | V62 | histidine kinase | quorum sensing, virulence, toxin production | (16, 17) |
| 3404503 | G8E05_16005 | ST19 | histidine kinase | quorum sensing, virulence, toxin production | (16, 17) |
| 3405031 | - | ST41 | intergenic region within *agr-2* region II | quorum sensing, virulence, toxin production | (16, 17) |
| 3406020 | G8E05_16020 | V73, ST7 | histidine kinase | quorum sensing, virulence, toxin production | (16, 17) |
| 3406111 | G8E05_16020 | ST39, ST40, ST43, ST44 | histidine kinase | quorum sensing, virulence, toxin production | (16, 17) |
| 3406139 | G8E05_16020 | ST21 | histidine kinase | quorum sensing, virulence, toxin production | (16, 17) |
| 3406863 | G8E05_16025 | ST34 | sensory transduction histidine kinase | quorum sensing, virulence, toxin production | (16, 17) |
| 3407428 | G8E05_16025 | H18, ST33 | sensory transduction histidine kinase | quorum sensing, virulence, toxin production | (16, 17) |
| 3407745 | G8E05_16025 | ST29 | sensory transduction histidine kinase | quorum sensing, virulence, toxin production | (16, 17) |
| 3407852 | G8E05_16025 | ST25 | sensory transduction histidine kinase | quorum sensing, virulence, toxin production | (16, 17) |
| 3407927 | G8E05_16025 | V41 | sensory transduction histidine kinase | quorum sensing, virulence, toxin production | (16, 17) |
| 3540160 | G8E05_16640 | ST39, ST40, ST43, ST44 | DegV family protein (start codon loss) | - | - |
| 3797980 | MnmE | ST44 | tRNA modification enzyme MnmE | virulence | (18) |
| 3833093 | G8E05_18150 | ST43 | MarR family transcriptional regulator | antibiotic resistance | (7) |
| 3833247 | G8E05_18150 | ST7B | MarR family transcriptional regulator | antibiotic resistance | (7) |

**References**

1. Lupien A, Gingras H, Leprohon P, Ouellette M. Induced tigecycline resistance in *Streptococcus pneumoniae* mutants reveals mutations in ribosomal proteins and rRNA. J Antimicrob Chemother. 2015;70(11):2973-80.

2. Deng CY, Deng AH, Sun ST, Wang L, Wu J, Wu Y, et al. The periplasmic PDZ domain-containing protein Prc modulates full virulence, envelops stress responses, and directly interacts with dipeptidyl peptidase of *Xanthomonas oryzae* *pv. oryzae*. Mol Plant Microbe Interact. 2014;27(2):101-12.

3. Schar J, Stoll R, Schauer K, Loeffler DI, Eylert E, Joseph B, et al. Pyruvate carboxylase plays a crucial role in carbon metabolism of extra- and intracellularly replicating *Listeria monocytogenes*. J Bacteriol. 2010;192(7):1774-84.

4. Kuenemann MA, Spears PA, Orndorff PE, Fourches D. In silico predicted glucose-1-phosphate uridylyltransferase (GalU) inhibitors block a key pathway required for *Listeria* virulence. Mol Inform. 2018;37(6-7):e1800004.

5. Tasteyre A, Barc MC, Collignon A, Boureau H, Karjalainen T. Role of FliC and FliD flagellar proteins of *Clostridium difficile* in adherence and gut colonization. Infect Immun. 2001;69(12):7937-40.

6. Ghelardi E, Celandroni F, Salvetti S, Beecher DJ, Gominet M, Lereclus D, et al. Requirement of *flhA* for swarming differentiation, flagellin export, and secretion of virulence-associated proteins in *Bacillus thuringiensis*. Journal of bacteriology. 2002;184(23):6424-33.

7. Grove A. MarR family transcription factors. Curr Biol. 2013;23(4):R142-R3.

8. Collins J, Robinson C, Danhof H, Knetsch CW, van Leeuwen HC, Lawley TD, et al. Dietary trehalose enhances virulence of epidemic *Clostridium difficile*. Nature. 2018;553(7688):291-4.

9. Lee JJ, Lee S-K, Song N, Nathan TO, Swarts BM, Eum S-Y, et al. Transient drug-tolerance and permanent drug-resistance rely on the trehalose-catalytic shift in *Mycobacterium tuberculosis*. Nat Comm. 2019;10(1):2928.

10. Park M, Mitchell WJ, Rafii F. Effect of trehalose and trehalose transport on the tolerance of *Clostridium perfringens* to environmental stress in a wild type strain and its fluoroquinolone-resistant mutant. Int J Microbiol. 2016;2016:4829716.

11. Willems RJ, Top J, Smith DJ, Roper DI, North SE, Woodford N. Mutations in the DNA mismatch repair proteins MutS and MutL of oxazolidinone-resistant or -susceptible *Enterococcus faecium*. Antimicrob Agents Chemother. 2003;47(10):3061-6.

12. Molle V, Fujita M, Jensen ST, Eichenberger P, González-Pastor JE, Liu JS, et al. The Spo0A regulon of *Bacillus subtilis*. Mol Microbiol. 2003;50(5):1683-701.

13. Turner AK, Lovell MA, Hulme SD, Zhang-Barber L, Barrow PA. Identification of *Salmonella typhimurium* genes required for colonization of the chicken alimentary tract and for virulence in newly hatched chicks. Infect Immun. 1998;66(5):2099-106.

14. Chastanet A, Derre I, Nair S, Msadek T. *clpB*, a novel member of the *Listeria monocytogenes* CtsR regulon, is involved in virulence but not in general stress tolerance. J Bacteriol. 2004;186(4):1165-74.

15. Selby K, Mascher G, Somervuo P, Lindström M, Korkeala H. Heat shock and prolonged heat stress attenuate neurotoxin and sporulation gene expression in group I *Clostridium botulinum* strain ATCC 3502. PLOS ONE. 2017;12(5):e0176944.

16. Cooksley CM, Davis IJ, Winzer K, Chan WC, Peck MW, Minton NP. Regulation of neurotoxin production and sporulation by a putative *agrBD* signaling system in proteolytic *Clostridium botulinum*. Appl Environ Microbiol. 2010;76(13):4448-60.

17. Ohtani K, Yuan Y, Hassan S, Wang R, Wang Y, Shimizu T. Virulence gene regulation by the *agr* system in *Clostridium perfringens*. J Bacteriol. 2009;191(12):3919-27.

18. Shippy DC, Fadl AA. tRNA modification enzymes GidA and MnmE: potential role in virulence of bacterial pathogens. Int J Mol Sci. 2014;15(10):18267-80.
