# Supplementary material for: Genomic and Phenotypic Characterization of Clostridium botulinum Isolates from an Infant Botulism Case Suggests Adaptation Signatures to the Gut
Source: mBio. 2022 May 2;13(3):e02384-21. doi: 10.1128/mbio.02384-21 (PMC9239077; doi:10.1128/mbio.02384-21)
Supplement: FIG S2 [file mbio.02384-21-s0003.pdf]

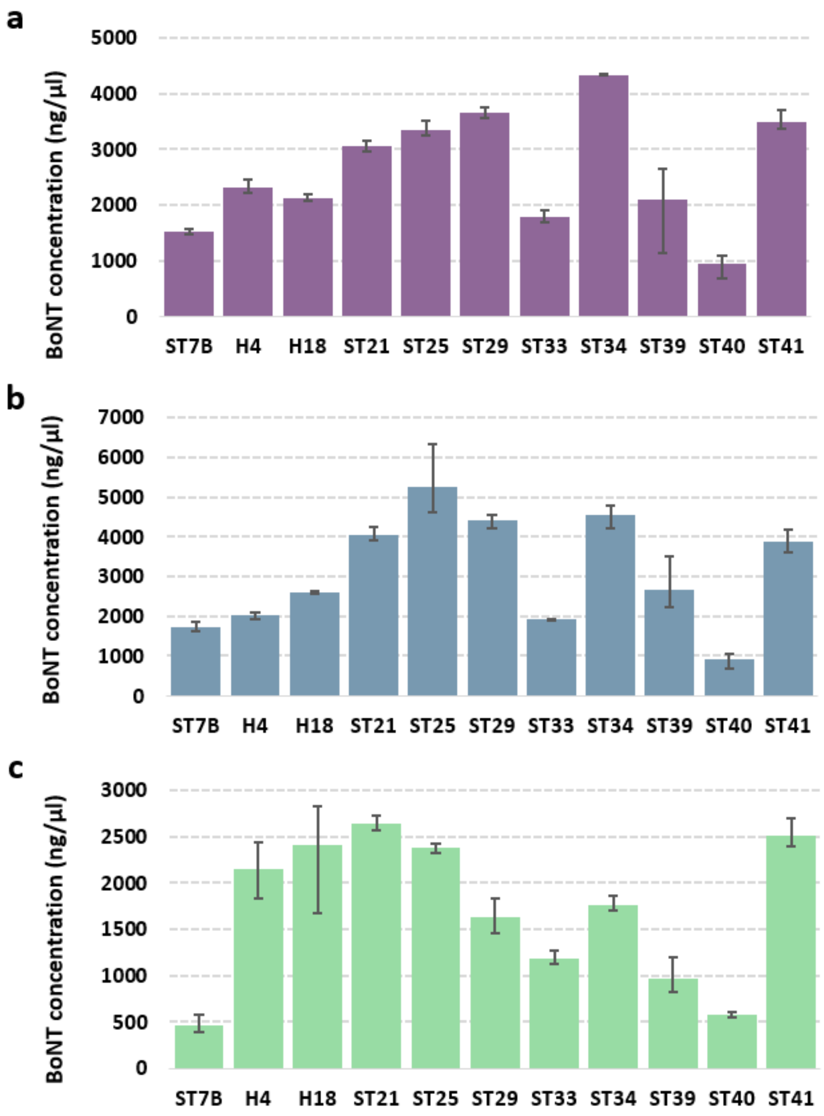

Figure S2. BoNT quantification using ELISA in *Clostridium botulinum* isolates after 24 hours. Bacteria grown in TYG (a), TPGY (b) and TPY (c). Error bars represent the minimum and maximum values obtained among replicates (N= 3). We selected a subset of isolates for BoNT quantification due to the presence of mutations in genes/operons that may be relevant in toxinogenesis, *i.e. agr-2* operon.
