# Supplementary material for: Genomic and Phenotypic Characterization of Clostridium botulinum Isolates from an Infant Botulism Case Suggests Adaptation Signatures to the Gut
Source: mBio. 2022 May 2;13(3):e02384-21. doi: 10.1128/mbio.02384-21 (PMC9239077; doi:10.1128/mbio.02384-21)
Supplement: FIG S3 [file mbio.02384-21-s0004.pdf]

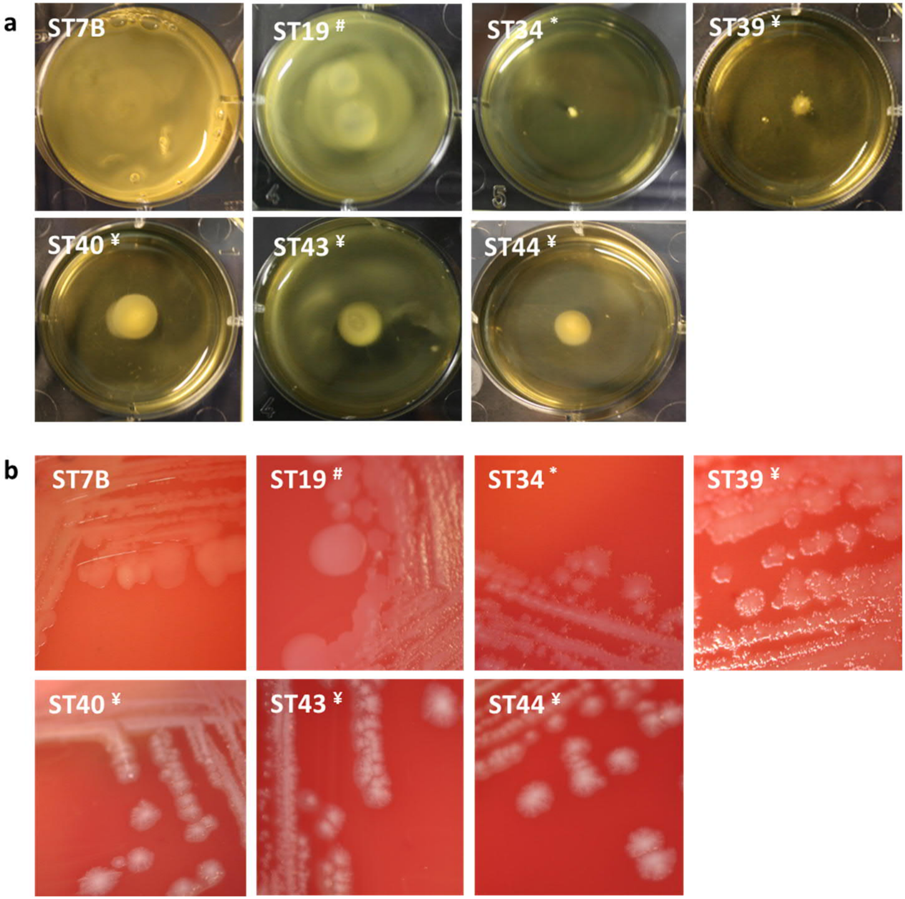

Figure S3. Motility phenotype (a) and colony morphology (b) of *Clostridium botulinum* isolates ST7B, ST19, ST34, ST39, ST40, ST43 and ST44. Motility assays were done in triplicate. Legend: # missense mutation in FlaA (Gln255Lys); * non-sense mutation in FlhA (Gln327*) and ¥ frame-shift insertion in FlgN (Glu3fs).
