# Supplementary material for: Genomic and Phenotypic Characterization of Clostridium botulinum Isolates from an Infant Botulism Case Suggests Adaptation Signatures to the Gut
Source: mBio. 2022 May 2;13(3):e02384-21. doi: 10.1128/mbio.02384-21 (PMC9239077; doi:10.1128/mbio.02384-21)
Supplement: TABLE S3 [file mbio.02384-21-s0008.pdf]

Table S3. Colony morphotype and motility phenotype of all *Clostridium botulinum* isolates.

Isolate	Origin	Colony morphotype	Motility [a]	Flagellar associated mutations
H4	household dust	smooth	n.d.	none
H18	household dust	smooth	n.d.	none
V41	household dust	smooth	n.d.	none
V62	household dust	smooth	n.d.	none
V134	household dust	smooth	n.d.	none
V206	household dust	smooth	n.d.	none
V1	household dust	smooth	n.d.	none
V4	household dust	smooth	n.d.	none
V73	household dust	smooth	n.d.	none
ST25	infant stool	smooth	n.d.	none
ST7B	infant stool	smooth	+	none
ST19	infant stool	smooth	+	FlaA (Gln255Lys)
ST4	infant stool	smooth	n.d.	none
ST7	infant stool	smooth	n.d.	none
ST21	infant stool	smooth	n.d.	none
ST29	infant stool	smooth	n.d.	none
ST31	infant stool	smooth	n.d.	none
ST32	infant stool	smooth	n.d.	none
ST33	infant stool	smooth	n.d.	none
ST34	infant stool	rough	-	FlhA (Gln327*)
ST39	infant stool	rough	+/-	FlgN (Glu3fs)
ST40	infant stool	rough	+/-	FlgN (Glu3fs)
ST41	infant stool	smooth	n.d.	none
ST43	infant stool	rough	+/-	FlgN (Glu3fs)
ST44	infant stool	rough	+/-	FlgN (Glu3fs)

[a] +, motile; -, non-motile; +/-, reduced motility; n.d., not determined; *, stop codon; fs, frame-shift mutation.
