# Supplementary material for: Genomic and Phenotypic Characterization of Clostridium botulinum Isolates from an Infant Botulism Case Suggests Adaptation Signatures to the Gut
Source: mBio. 2022 May 2;13(3):e02384-21. doi: 10.1128/mbio.02384-21 (PMC9239077; doi:10.1128/mbio.02384-21)
Supplement: FIG S4 [file mbio.02384-21-s0005.pdf]

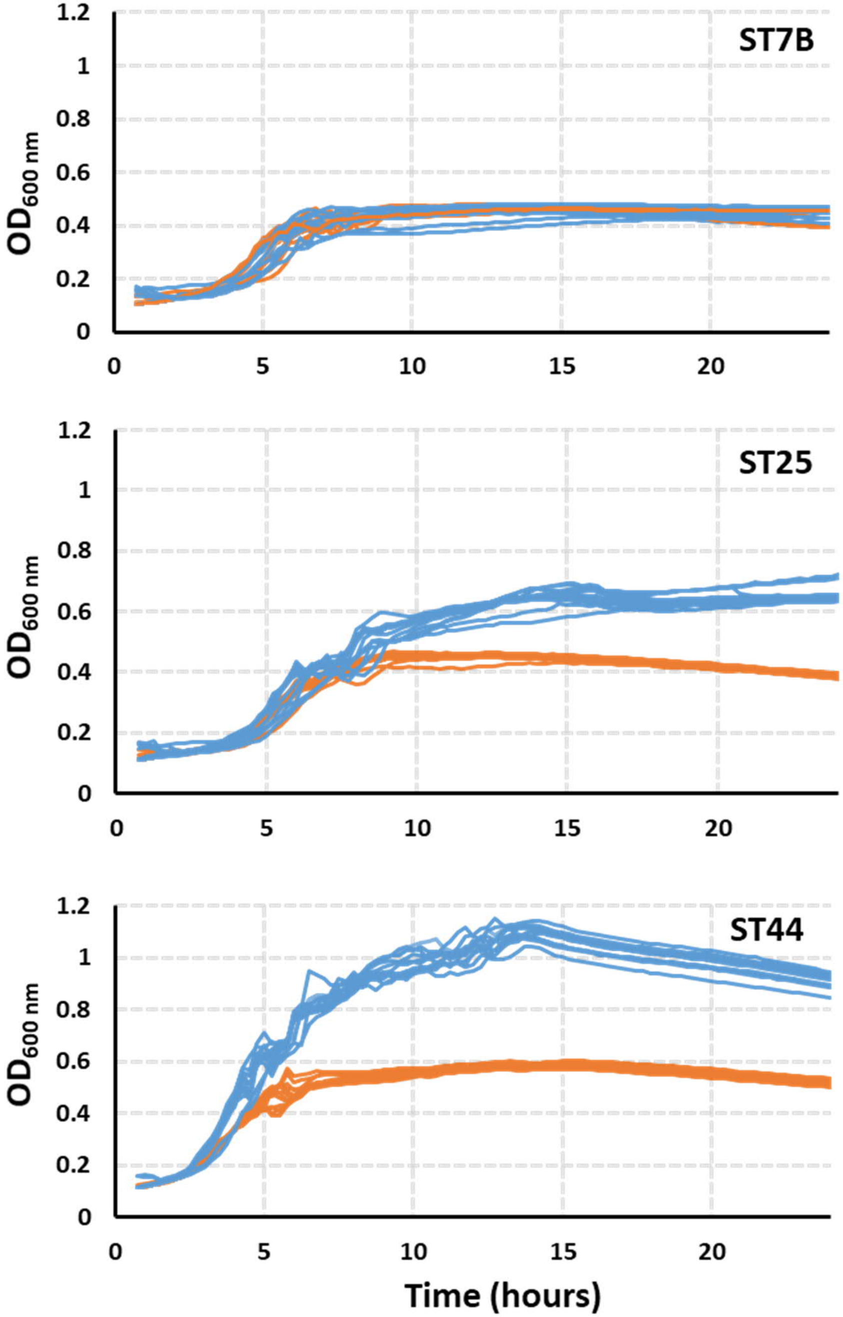

Figure S4. Growth curves of *C. botulinum* isolates ST7B, ST25 and ST44 grown for 24 hours in CDM-I medium (orange curves) and CDM-I medium supplemented with D-trehalose (blue curves). For each condition of each isolate, 12 replicates were made (four technical replicates originated from three biological replicates).
