# Supplementary material for: Genomic and Phenotypic Characterization of Clostridium botulinum Isolates from an Infant Botulism Case Suggests Adaptation Signatures to the Gut
Source: mBio. 2022 May 2;13(3):e02384-21. doi: 10.1128/mbio.02384-21 (PMC9239077; doi:10.1128/mbio.02384-21)
Supplement: TABLE S4 [file mbio.02384-21-s0009.pdf]

Table S4. Antibiotic susceptibility assay – determination of MIC (µg/ml) in *Clostridium botulinum* isolates. For comparison, ST7B (reference early stool isolate) and ST41 (late stool isolate) were included along with ST25 (early stool isolate with *treP* variant) and ST44 (late stool isolate with *treP* variant). The table shows the minimum and maximum values recorded among replicates.

Isolate	MX	LE	CI
ST7B	0.094-0.125	0.094-0.19	0.064-0.19
ST25	0.125-0.19	0.125-0.25	0.125-.019
ST41	0.125-0.19	0.125-0.19	0.064-0.25
ST44	0.125-0.25	0.125-0.25	0.064-0.19

MX, moxifloxacin; LE, levofloxacin; and CI, ciprofloxacin.
